# Transcription profiles of boron-deficiency-responsive genes in citrus rootstock root by suppression subtractive hybridization and cDNA microarray

**DOI:** 10.3389/fpls.2014.00795

**Published:** 2015-01-28

**Authors:** Gao-Feng Zhou, Yong-Zhong Liu, Ou Sheng, Qing-Jiang Wei, Cheng-Quan Yang, Shu-Ang Peng

**Affiliations:** ^1^Key Laboratory of Horticultural Plant Biology (Ministry of Education), College of Horticulture and Forestry Science, Huazhong Agricultural UniversityWuhan, China; ^2^National Navel Orange Engineering Research Center, College of Navel Orange, Gannan Normal UniversityGanzhou, China; ^3^Institute of Fruit Tree Research, Guangdong Academy of Agricultural SciencesGuangzhou, China; ^4^College of Agricultural, Jiangxi Agricultural UniversityNanchang, China

**Keywords:** citrus rootstock, boron deficiency, root, gene expression, SSH, cDNA microarray

## Abstract

Boron (B) deficiency has seriously negative effect on citrus production. Carrizo citrange (CC) has been reported as a B-deficiency tolerant rootstock. However, the molecular mechanism of its B-deficiency tolerance remained not well-explored. To understand the molecular basis of citrus rootstock to B-deficiency, suppression subtractive hybridization (SSH) and microarray approaches were combined to identify the potential important or novel genes responsive to B-deficiency. Firstly four SSH libraries were constructed for the root tissue of two citrus rootstocks CC and Trifoliate orange (TO) to compare B-deficiency treated and non-treated plants. Then 7680 clones from these SSH libraries were used to construct a cDNA array and microarray analysis was carried out to verify the expression changes of these clones upon B-deficiency treatment at various time points compared to the corresponding controls. A total of 139 unigenes that were differentially expressed upon B-deficiency stress either in CC or TO were identified from microarray analysis, some of these genes have not previously been reported to be associated with B-deficiency stress. In this work, several genes involved in cell wall metabolism and transmembrane transport were identified to be highly regulated under B-deficiency stress, and a total of 23 metabolic pathways were affected by B-deficiency, especially the lignin biosynthesis pathway, nitrogen metabolism, and glycolytic pathway. All these results indicated that CC was more tolerant than TO to B-deficiency stress. The B-deficiency responsive genes identified in this study could provide further information for understanding the mechanisms of B-deficiency tolerance in citrus.

## Introduction

Abiotic stress, such as nutrient deficiency or toxicity, drought, high salinity, extreme temperature, and flooding is a major cause of crop loss worldwide, reducing average yields for most major crop plants by more than 50% (Bray et al., 2000). It is well-known that boron (B) is an essential micronutrient required for plant growth and development, which affects not only yield but also the quality of crops (Warington, 1923; Brown et al., 2002). However, B-deficiency is frequently observed because the boric acid in soil is easily leached under high rainfall conditions. Worldwide, B-deficiency is more extensive than deficiency of any other plant micronutrient, and it is one of the major constraints to 132 crops production in many parts of the world (Shorrocks, 1997). In fact, B nutrition has been related with changes in phenolic metabolism (Camacho-Cristóbal et al., 2002), membrane integrity and function (Cakmak and Römheld, 1997), nitrate assimilation (Camacho-Cristóbal and González-Fontes, 1999, 2007), and cell wall synthesis and structure (Kobayashi et al., 1996), among others (Blevins and Lukaszewski, 1998; Brown et al., 2002). In plant cell wall, B can cross-link pectic polysaccharides through borate-diol bonding of two rhamnogalacturonan II (RG-II) molecules (Ishii and Matsunaga, 1996; Kobayashi et al., 1996; O'Neill et al., 1996, 2004), and B has been shown to be essential to the structure and function of plant cell walls (O'Neill et al., 2004). Hence, previous physiological studies on the effects of B-deficiency to the root elongation (Kouchi and Kumazawa, 1975), leaf expansion (Dell and Huang, 1997), viable pollen grain production, and pollen tube elongation (Cheng and Rerkasem, 1993) were conducted.

During the past 20 years, more and more works were carried and a greatly advanced knowledge of B transport mechanisms at the molecular level has been achieved (Takano et al., 2008). Two different types of transporters were identified as B transport molecules required for efficient B translocation under B-limited conditions in *Arabidopsis thaliana* (Takano et al., 2002, 2006). The first type B transporter is B special exporter. In this type BOR1 was first reported in *A. thaliana* (Takano et al., 2002). *Arabidopsis bor1-1* mutants are highly sensitive to B-deficiency (Noguchi et al., 1997, 2000). As a B exporter BOR1 is required for efficient xylem loading and preferential translocation of B into young portions of plants under B-deficient conditions (Noguchi et al., 1997; Takano et al., 2001, 2002, 2005). Aquaporins are the second type B transporter. Aquaporins are water channel proteins of intracellular and plasma membranes that mediate the transport of water and/or small neutral solutes (Maurel, 2007; Maurel et al., 2008). Based on sequence homology and localization, plant aquaporins can be subdivided into four subgroups: small basic intrinsic proteins (SIPs), nodulin-26-like intrinsic protein (NIPs), tonoplast intrinsic protein (TIPs) and plasma membrane intrinsic proteins (PIPs). Among them, NIP5;1 is a member of the major intrinsic protein (MIP) family and has been identified as a boric acid channel required for plant growth under low B conditions in *A. thaliana* (Takano et al., 2006). NIP5;1 is a plasma membrane boric acid transporter expressed in root epidermal, cortical, and endodermal cells. Expression of the *NIP5;1* transcript is up-regulated in response to B deprivation. NIP5;1 is involved in B uptake from the root surface under conditions of B limitation as a major boric acid channel (Takano et al., 2006). *NIP6;1*, is the most similar gene to *NIP5;1* in *Arabidopsis* and both belong to NIP subgroup II. NIP6;1 is a boric acid channel involved in preferential B transport to growing tissues of plants and showed the function of a boric acid channel in shoots in *Arabidopsis* (Tanaka et al., 2008). Transport of B to growing tissues of plants under B-deficient conditions occurs not only by apoplastic flow via the transpiration stream but also via other mechanisms, such as xylem–phloem transfer, which involve facilitated flux across the membranes of living cells. NIP6;1 is involved in this latter mechanism (Tanaka et al., 2008).

Citrus is one of the most important economic fruit crops in the word. As important rootstocks for the majority of the citrus, Trifoliate orange (TO) and Carrizo citrange (CC) are known for being widely used in China and other citrus cultivation regions of the world. However, compared with *Arabidopsis*, much less is known about the mechanism behind citrus plant responses to B-deficient stress. Although citrus plants are not classified as the most sensitive species to B-deficiency, the occurrence of B-deficiency has been reported in the major citrus producing countries of the world, such as Spain, United States, Brazil, and China (Shorrocks, 1997; Sheng et al., 2008, 2009). In eastern and southern china where is the major area of navel orange production, the soil B levels are low (hot water extraction B < 0.25 mg Kg^−1^) (Sheng et al., 2008). Soil fertilization with B is one approach to grow citrus plants undertaken to prevent B-deficiency in the field (Schon and Blevins, 1990). However, fertilization is costly and excess B is also toxic to plants (Nable et al., 1997). A narrow B concentration range exists between deficient and toxic level for plants, which complicates B fertilizer application (Francois, 1984; Schon and Blevins, 1990). Using the identified transporters, transgenic plants tolerant to low-B conditions have been generated by artificially up-regulating expression of B transporter in *A. thaliana* plants. Overexpression of *AtNIP5;1*, a boric acid channel gene for root B uptake, and/or *AtBOR1*, an efflux B transporter gene for xylem loading, improves the vegetative and reproductive growth of *A. thaliana* under B-deficient condition (Miwa et al., 2006; Kato et al., 2009). The generation of B-deficiency-tolerant *A. thaliana* plants suggests that up-regulating B-transporter expression can improve the growth of crops under B-deficiency conditions. Such as, overexpression of an *A. thaliana* borate transporter *AtBOR1* gene improved growth in tomato under B-deficient conditions (Uraguchi et al., 2014). Thus, understanding the B transport mechanisms is important to improve B nutrition of citrus. Previous works have suggested that Carrizo citrange [*Citrus sinensis* (L.) Osb. × *Poncirus trifoliata* (L.) Raf.] (abbreviated as CC) is a tolerant rootstock and TO [*P. trifoliata* (L.) Raf.] (abbreviated as TO) is a sensitive rootstock to B-deficiency (Sheng et al., 2009; Mei et al., 2011; Zhou et al., 2014). However, little is known about the molecular basis of the different phenotypes to B-deficiency. In this study, to understand the molecular basis of citrus rootstock to B-deficiency, suppression subtractive hybridization (SSH) and microarray approaches were combined to identify differentially expressed genes in CC and TO. Four SSH libraries were constructed for the root tissue of two citrus rootstocks CC and TO to compare B-deficiency treatment and non-treatment plants.

## Materials and methods

### Plant materials and B-deficiency treatments

Two navel orange rootstocks, CC [*C. sinensis* (L.) Osb. × *P. trifoliata* (L.) Raf.] and TO [*P. trifoliata* (L.) Raf.], were used in this experiment. Seeds of these two rootstocks were surface sterilized in a 5% (v/v) hypochlorite solution for 15 min and then washed 3 times in 70% (v/v) ethanol and 3 times in sterile H_2_O. These seeds were placed on a porcelain tray with moistened gauze and transferred to an incubator at 30°C, then they were moistened every day with sterile water till seed germination. The seeds germinated at 10–15 days were selected and than transferred into 14 L plastic pots filled with vermiculite, 20 plants in each pot. Experiments were carried out in a growth chamber with a light/dark regime of 14/10 h, 28/22°C, 75% relative humidity and light intensity of 800 μmol m^−2^ s^−1^ of photosynthetically active radiation. Irrigating them twice a week, until the plants have four leaves (about 3 weeks later). Then they were selected by uniform size and transferred into hydroponics with 4 L solution. The plants were pre-cultured with 1/2 strength Hoagland's No. 2 nutrient solution for 3–4 weeks, until the new white root appeared. The 1/2 strength Hoagland's No. 2 nutrient solution contained 6 mM KNO_3_, 4 mM Ca(NO_3_)_2_, 1 mM NH_4_H_2_PO_4_, 2 mM MgSO_4_, 9 μM MnCl_2_, 15 μM H_3_BO_3_ 0.8 μM ZnSO_4_, 0.3 μM CuSO_4_, 0.01 μM H_2_MoO_4_, and 50 μM Fe-EDTA (Hoagland and Arnon, 1950). The solution was ventilated for 20 min every 2 h and replaced twice a week. The pH of all nutrient solutions were adjusted to 6.0 with 0.1 M KOH. For investigating the physiological and morphological changes of CC and TO in response to B-deficiency, the pre-cultured plants in normal solution were transferred either into a new nutrient solution with 0.01 mg L^−1^ B as B-deficiency treatment or with 0.25 mg L^−1^ B as control for 8 weeks. While for SSH cDNA libraries and microarray analysis, the plant samples were harvested at 3, 6, 12, 24, 48 and 96 h after treatment.

### Determination of root B-concentration and root morphology

After 8 weeks, three biological replicates (six seedling plants per replicate) were harvested randomly for each treatment, and rinsed with deionised water. Then the seedlings were divided into leaf, stem and root. Root samples were scanned with an Epson digital scanner (Expression 10000XL 1.0, Epson Inc. Japan) and the image was analyzed by WinRhizo Pro (S) v. 2009c (Regent Instruments Inc., Canada) software for root morphology, including total root length, root surface area, root volume, and root number. After root morphological analysis, the fresh roots were placed into a forced air oven at 105°C for 15 min, and then at 75°C until constant weight were reached to determine the root dry weight. All the dried samples were ground into fine powder for determination the B concentration in root following the method described by Storey and Treeby (2000). Briefly, 0.50ġ of each root sample were dry-ashed in a muffle furnace at 500°C for 6 h, followed by dissolution in 0.1 N HCl, and B concentration was determined using inductively coupled plasma atomic spectroscopy (ICP-AES; Thermo Inc, IRIS Advan, USA).

### Total RNA extraction and mRNA isolation

Root samples were harvested at six time points (3, 6, 12, 24, 48 and 96 h after treatment), three biological replicates (24 seedling plants per replicate) were harvested randomly and frozen immediately in liquid nitrogen for RNA isolation. Total root RNA was isolated by TriZOL reagent (Takara, Japan) from each time point. For SSH, equal amounts of total RNA for each sample from treatment or control were mixed and the mRNA was purified from the mixed total RNA using the Oligotex mRNA Mini Kit (Qiagen, Germany) according to the manufacturer's protocol. The total RNA and mRNA were quantified spectrophotometrically at wavelengths of 230, 260, and 280 nm, and mRNA was adjusted to a final concentration of 0.5 μg μl^−1^. The integrity of the total RNA and mRNA was verified by subjecting samples to electrophoresis on 1.2% agarose gels.

### Construction of SSH cDNA libraries and amplification of cDNA inserts

The cDNA reversely transcribed from 2 μg of the mixed mRNA mentioned above was used for SSH with the Clontech PCR Select-cDNA Subtraction Kit (BD Biosciences Clontech, Palo Alto, CA, USA). Both forward and reverse SSH libraries of CC and TO were constructed following the manufacturer's protocol, respectively. The brief protocol was described by Ouyang et al. (2007).

Individual bacterial clones containing 9216 citrus uniEST from the library were randomly chosen and distributed into 384-well plates. These clones were cultured overnight at 37°C and used as PCR templates. PCR amplification was conducted following Shi et al. (2006). Aliquots (1 μl) of the PCR reactions were analyzed in a 0.8% agarose gel and examined by Bio-Rad UV spectroscopy (Bio-Rad Laboratories, Washington, DC, USA) to ensure both the quality and quantity. The remaining cDNA was precipitated with addition of 260 μl anhydrous ethanol-sodium acetate (25:1) and resuspended in 30 μl sterile water.

### cDNA microarray slides preparation

The PCR products were precipitated again by addition of 100 μl anhydrous ethanol and resuspended in 15 μl 50% DMSO at a final concentration of 0.1–0.5 μg μl^−1^ and then spotted onto amino silaned glass slides (CapitalBio. Corp., Beijing, China) with a SmartArraver™ microarrayer (CapitalBio Corp., Beijing, China). Each clone was printed in triplicate. After printing, the slides were baked for 1 h at 80°C and stored dry at room temperature till use. Prior to hybridization, the slides were rehydrated over 65°C water for 10 s, snap dried on a 100°C heating block for 5 s, and UV cross-linked at 250 mJ cm^−2^. The unimmobilized PCR products were washed off with 0.5% SDS for 15 min at room temperature and SDS was removed by dipping the slides in anhydrous ethanol for 30 s. The slides were spin-dried at 1000 rpm for 2 min. Eight sequences derived from intergenic regions in yeast genome, showing no significant homology to all the existing sequences in GenBank, were spotted multiple times onto the microarray as exogenous controls. Total citrus RNA was spiked with a mixture of these exogenous control RNAs to validate the semi-quantitative microarray result.

### Preparation of fluorescent dye-labelled cDNA and hybridization

The gene expression profiles in root tissue after 6, 12, and 24 h severe B-deficiency stress and the corresponding controls were investigated by microarray analysis. An aliquot of 5 μl total RNA was used to produce CY5/CY3-labeled cDNA employing an RNA amplification combined with Klenow enzyme labeling strategy according to a previous published protocol (Guo et al., 2005). Cy5/Cy3-labeled cDNA was hybridized with the microarray at 42°C overnight. Each hybridization was performed in duplicate by dye swap. After that, the arrays were washed with 0.2% SDS, 2× SSC at 42°C for 5 min, and then with 0.2% SSC for 5 min at room temperature.

### Microarray data and EST sequence analysis

Arrays were scanned with a confocal laser scanner, LuxScan™ 10 K (CapitalBio Corp., Beijing, China), and the resulting images were analyzed with SpotData Pro 2.0 software (CapitalBio Corp.). Spots with fewer than 50% of the signal pixels exceeding the local background value for both channels (Cy3 and Cy5) plus two standard deviations of the local background were removed. cDNA spots with less than four out of a total of six data points in each replicated hybridization were removed. A spatial and intensity dependent (LOWESS) normalized ratio data were then log transformed. Differentially expressed genes were identified using a *t*-test, and multiple test corrections were performed using false discovery rate (FDR) (Benjamini and Hochberg, 1995). Genes with FDR< 0.01 and a fold change ≥ 2 were identified as differentially expressed genes.

All the clones differentially expressed after 12 h B-deficiency treatment were subjected to single-pass sequencing reaction from the 5′ end (BIG, Wuhan, China). Low quality regions, vector and adaptor sequences were removed using LUCY program (Chou and Holmes, 2001). The remaining ESTs were compared with the GenBank database using BLASTx (http://www.ncbi.nlm.nih.gov/BLAST/), with 10^−5^ as the cutoff e-values, and annotated after the homologous sequence in GenBank. All unigenes described in the present paper have been submitted to GenBank with the accession numbers JK817580 to JK817718.

### Quantitative real-time PCR verification

Total root RNA was isolated from three stages (6, 12, and 24 h) of both stressed and control plants were performed as describing above. First strand cDNA was synthesized from 8 μg total RNA from each sample using MMLV reverse transcriptase (Toyobo, Osaka, Japan) according to the supplier's manual, Primer pairs were designed with the Primer Express software (Applied Biosystems, Foster city, CA, USA). Primer sequences are provided in Table S1. Real-time PCR verification was performed according to Qiu et al. (2012).

### Histochemical staining and microscopy

Histochemical localization of the lignin was done using phloroglucinol. Using approximately 20 μm-thick hand-cut sections from the root of CC and TO. The stained sections were examined and photographed with a light microscope (Nikon Eclipse E600) for lignin (phloroglucinol). For scanning electron microscopy, the root tissues were excised and fixed in 2.5% (v/v) glutaraldehyde. The fixed samples were washed twice in 0.1 M sodium cacodylate buffer for 15 min each, postfixed in 1% OsO4 for 1 h, dehydrated through an ethanol gradient, and infiltrated. Samples were critical point dried, sputter coated with gold in an E-100 ion sputter, and viewed with a scanning electron microscope (Carl Zeiss EV040). For transmission electron microscopy, ultrathin sections were made using an ultramicrotome (MT-X; RMC), and the sections were thoroughly stained with aqueous 2% uranyl acetate for 10 min followed by lead citrate for 2 min. The sections were viewed with a JEM-1010 electron microscope (JEOL) operating at 60 kV.

## Results

### The differential performance of CC and to under B-deficiency

**Figure 1:**
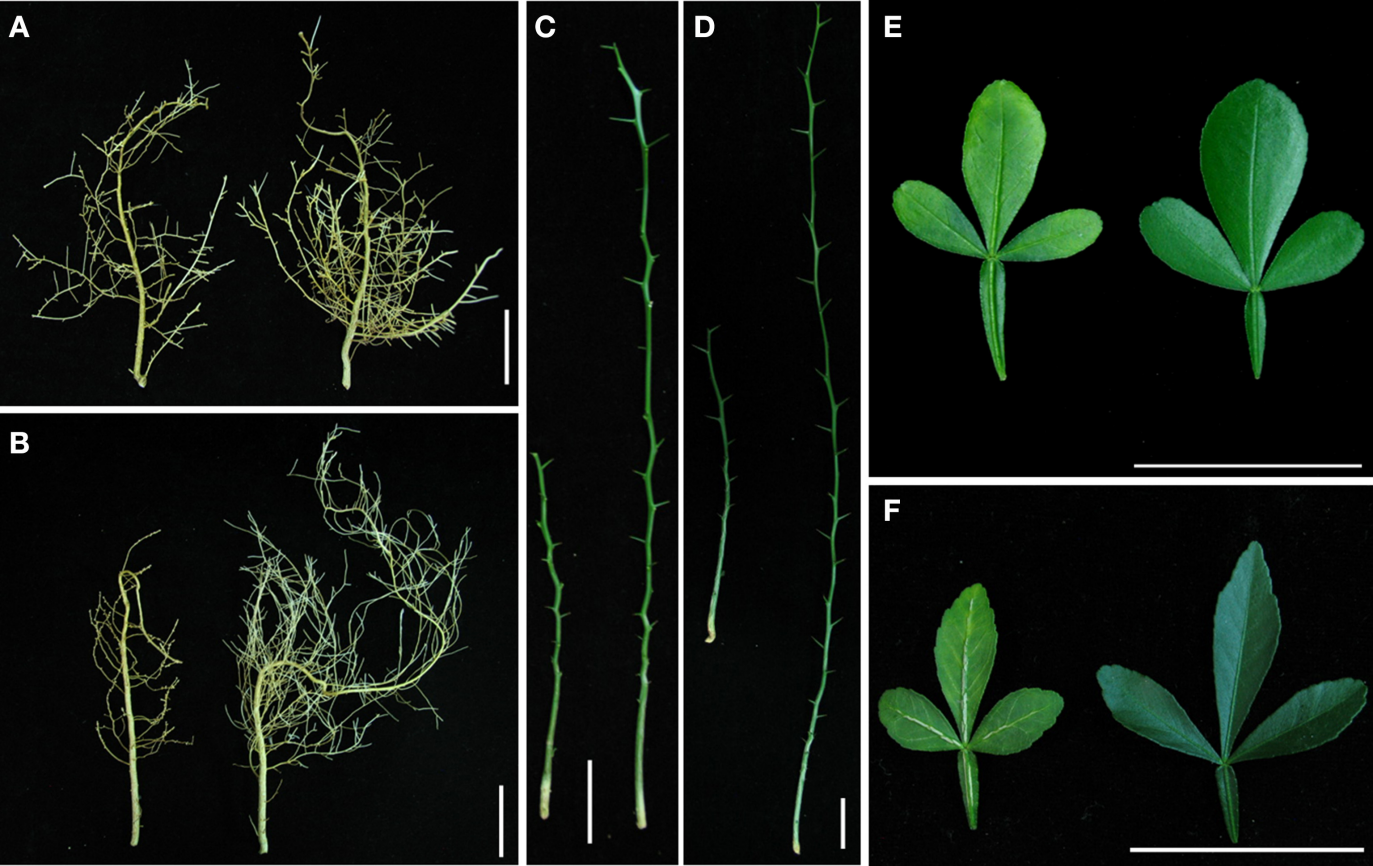
**The different performance of Carrizo citrange (CC) and Trifoliate orange (TO) seedlings under boron (B)-deficiency stress for 8 weeks**. 2-month-old plants were grown in hydroponics and treated for another 8 weeks. **(A)** Root of CC under B-deficiency stress (left) and control (right); **(B)** Root of TO under B-deficiency stress (left) and control (right); **(C)** Stem of CC under B-deficiency stress (left) and control (right); **(D)** Stem of TO under B-deficiency stress (left) and control (right); **(E)** Leaf of CC under B-deficiency stress (left) and control (right); **(F)** Leaf of TO under B-deficiency stress (left) and control (right).

After 0.01 mg L^−1^ B treatment for 8 weeks, differential performance of CC and TO was observed in different plant parts. Vein swelling or cracking was observed in the leaves of TO under B-deficiency conditions, whereas no significant visual symptoms were detected in CC, except for a slight yellowing found in several leaves at the end of the experiment (Figures 1E,F). Due to the shoot tip necrosis, the length of stem was decreased markedly in both CC and TO under B-deficiency conditions (Figures 1C,D). The most dramatic morphological difference was found in root of TO between normal and B-deficiency treatment. The lateral roots of TO were longer under normal conditions, but they were shorter and thicker under B-deficiency conditions (Figure 1B); However, no significant difference was found in CC (Figure 1A).

**Figure 2:**
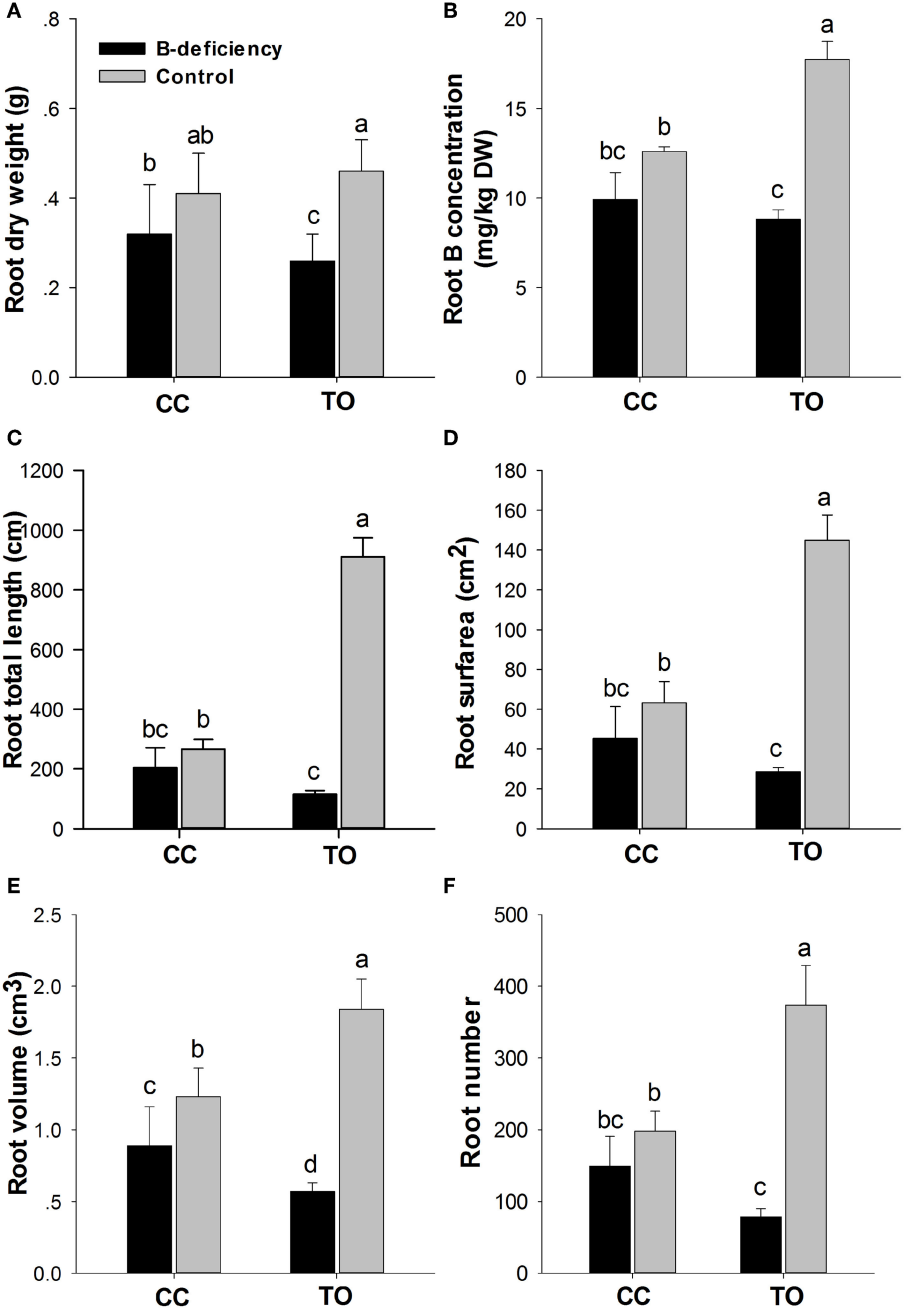
**Phenotypic responses of Carrizo citrange and Trifoliate orange root by boron-deficient stress**. 2-month-old plants were grown in hydroponics and treated for another 8 weeks. After that root dry weight **(A)** and B concentration in root **(B)** were measured. Moreover, the root morphologies of root total length **(C)**, root surface **(D)**, root volume **(E)** and root number **(F)** were also measured. Data are presented as mean ± SE of three biological replicates (*n* = 6), samples from six plants were collected for each biological replicate. Different letters indicate significant differences (*p* < 0.05) between the different genotypes and different treatments.

The root dry weight was dramatically decreased in TO, but only a small decrease was observed in CC under B-deficiency when compared to control (Figures 1A,B, 2A). In addition, B concentration in the root of TO was significantly reduced under B-deficiency treatment, whereas no effect was found in the root of CC. It is worthy to note that the concentration in the root of CC was lower than in TO under normal conditions (Figure 2B). The root morphology of these two rootstocks was also analyzed under B-deficiency conditions. The root length, root surface area, root volume and root number were decreased markedly in response to B-deficient stress in TO. However, only the root volume was decreased significantly in CC under B-deficient conditions and no significant decrease was found for other three parameters (Figures 2C–F). All these results supported that CC was more tolerant to B-deficiency than TO.

### SSH libraries construction and overall features of the B-deficiency responsive expression profile

**Figure 3:**
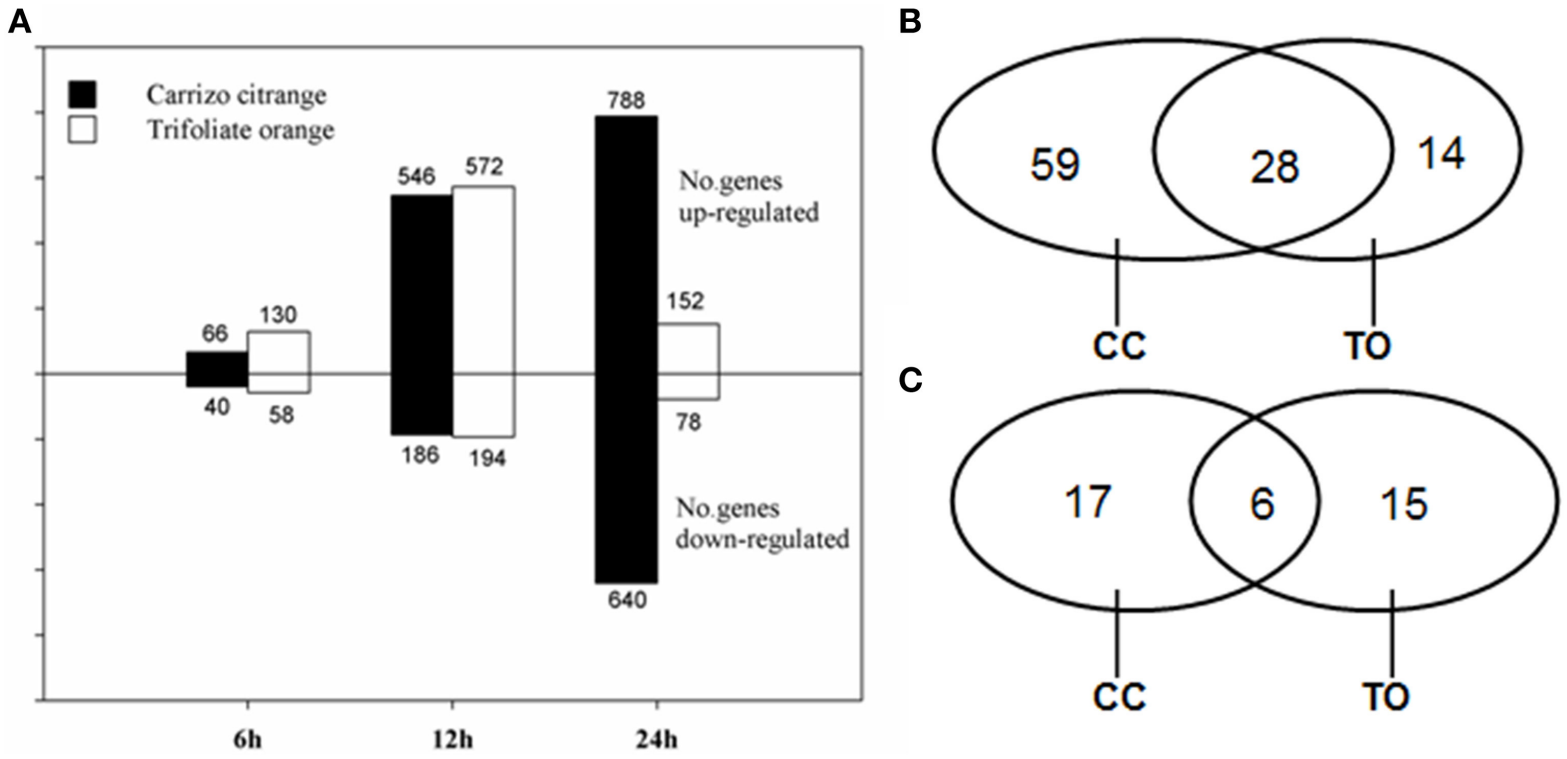
**Number of differentially expressed clones/genes. (A)** Number of SSH cDNA clones significantly up- or down-regulated in Trifoliate orange and Carrizo citrange in response to boron deficient stress at various time points. **(B,C)** Venn diagram illustrates the number of unique genes up- **(B)** or down-regulated **(C)** by boron deficient stress in either or both rootstocks.

Forward and reverse subtractions were conducted between root tissues from B-deficiency stress and non-stress CC and TO plants, respectively. One thousand nine hundred and twenty clones were randomly picked from each SSH library. In total, 7680 clones from the four SSH libraries were amplified and used for microarray analysis. The insert size of the SSH clones was from 0.45 to 0.75 kb, and most of them were around 0.55 kb. RNA samples from the root tissues at the stages of 6, 12, and 24 h after B-deficiency stress and the same time points of non-treated control plants were used for microarray hybridization. In total, 2266 and 1184 differentially expressed cDNA clones (FDR <0.01 and fold change ≥ 2) from either CC or TO were identified under B-deficiency conditions, respectively. As shown in Figure 3A, the expression patterns of CC and TO were similar at 6 and 12 h, but distinct at 24 h. At 6 and 12 h time point, there was no significant difference between CC and TO; However, after 24 h treatment the number of differentially expressed cDNA clones of CC was 5.2 and 8.2 fold higher in up- and down-regulated genes, respectively, compared to TO.

All the differentially expressed clones at 12 h of B-deficiency stress were subjected to one single-pass sequencing (464 redundant clones). After removing low quality regions, vector and adaptor sequences, 409 high-quality ESTs were obtained. These clean EST high quality sequences were assembled into unigenes with CAP3 program, and 139 unique genes (45 contigs and 94 singletons) were obtained. Among the 45 contigs, most of them contained 2 or 3 ESTs, whereas only 9 contigs contained 4–9 ESTs. Among the 139 unique genes, 101 were up-regulated and 38 were down-regulated. In the up-regulated genes, 59 were only identified in CC, 14 were only identified in TO, and 28 were identified in both of them (Figure 3B). As for the down-regulated genes, 17 were from CC only, 15 were from TO only, and 6 were from both (Figure 3C). This detailed analysis also suggested a relatively larger number of genes changed significantly in the B-tolerant rootstock CC.

**Figure 4:**
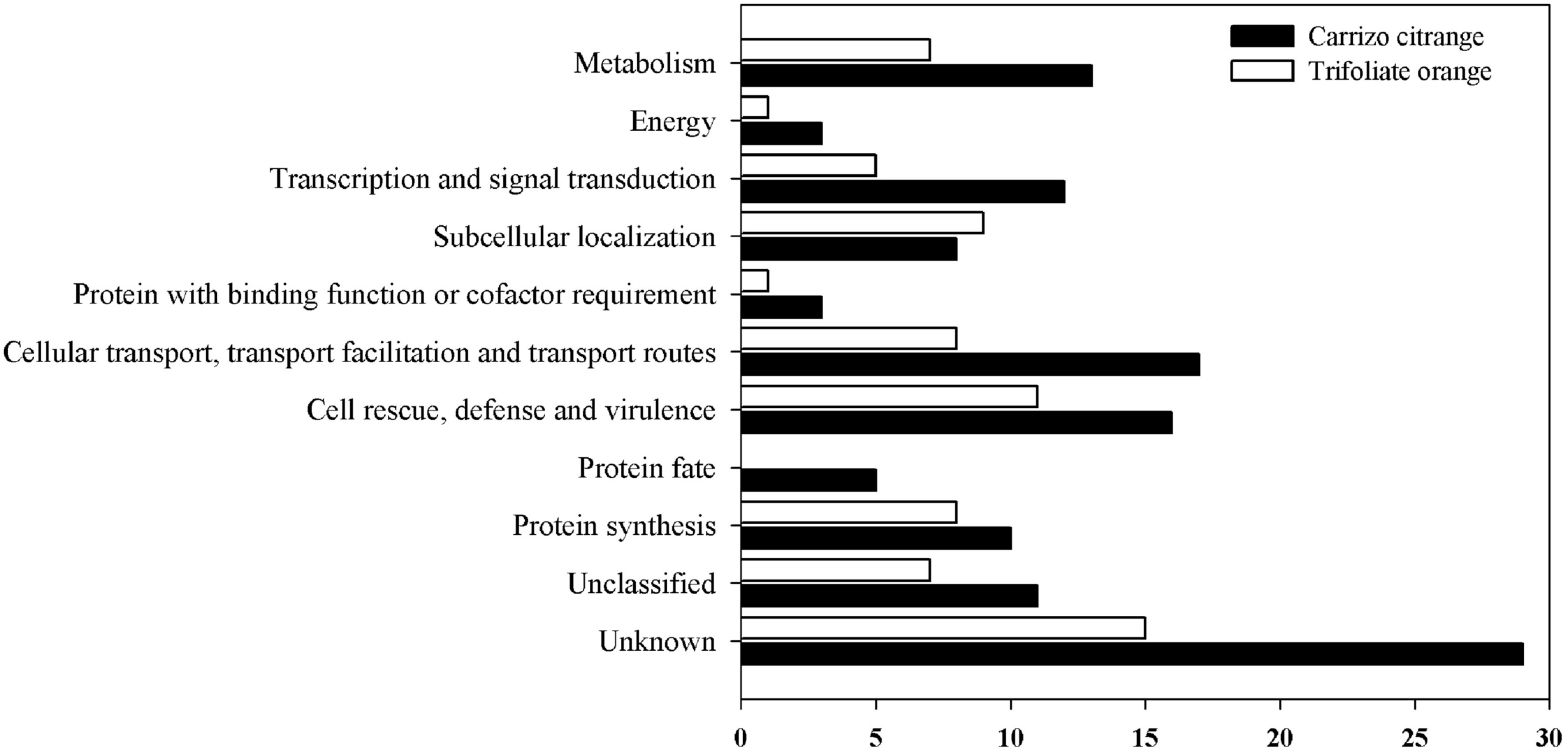
**Distribution of differentially expressed unique genes in two rootstocks, CC, and TO**. A total of 139 unique genes were grouped into 11 functional categories based on MIPS functional categories.

All these unique genes were functionally annotated by blasting against the GenBank non-redundant protein database, and subsequently submitted to GenBank with the accession numbers JK817580 to JK817718 (Table S2). Distribution of differentially expressed genes of citrus rootstocks are shown in Figure 4, a total of 139 unique genes were grouped into 11 functional categories based on MIPS functional categories. The number of differentially expressed genes was higher in CC than TO of all functional categories, except for the functional category of subcellular localization. The majority of differentially expressed genes in CC were involved in transport, cell rescue and defense, and metabolism. While in TO were involved in cell rescue and defense, subcellular localization, and protein fate.

### Verification of microarray data

**Figure 5:**
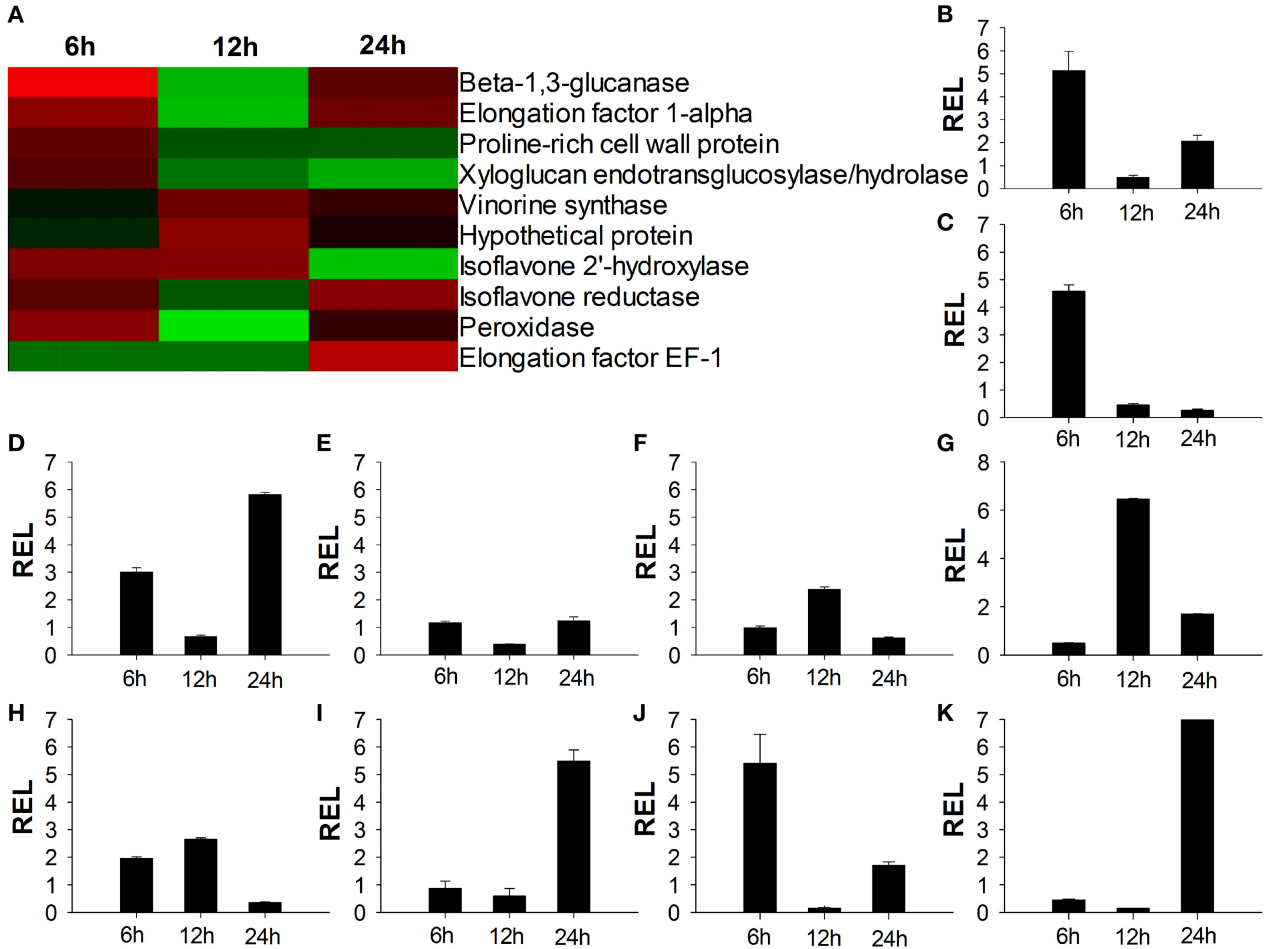
**Verification of microarray results by quantitative real-time RT-PCR. (A)** Expression ratios of the selected genes from microarray analysis results; **(B–K)** Quantitative real-time RT-PCR analysis of the selected genes. The relative expression level of Beta-1,3-glucanase gene **(B)** Elongation factor 1-alpha gene **(C)** Proline-rich cell wall protein gene **(D)** Xyloglucan endotransglucosylase/hydrolase gene **(E)** Vinorine synthase gene **(F)** Hypothetical protein gene **(G)** Isoflavone 2′-hydroxylase gene **(H)** Isoflavone reductase gene **(I)** Peroxidase gene **(J)** Elongation factor EF-1 gene **(K)**. RNA of seedling root was extracted at three time points and gene expression levels were analyzed by Quantitative real-time RT-PCR assays Data presented are means ± SE of three biological replicates (*n* = 6). REL, Relative expression level.

To confirm the results obtained using cDNA microarray analysis, 10 genes were randomly selected to analyze their expression profiles by quantitative real-time PCR. Quantitative real-time PCR was performed using total RNA isolated from 6 to 24 h in both CC and TO plants root, respectively. The gene-specific primer pairs are listed in Table S1. Quantitative real-time PCR data agreed with the microarray data for 27 out of 30 (90%) data points (Figure 5). These results confirmed the differential expression of all 10 selected genes.

### Cell wall genes were highly changed under B-deficiency stress

Several genes involved in cell wall metabolism were identified to be highly regulated under B-deficiency stress (Table 1). These genes include four xyloglucan endotransglycosylase/hydrolase 9 genes (*XTH9*; JK817598, JK817599, JK817606, and JK817615), two proline-rich cell wall protein 2 (*PRP2*; JK817586 and JK817604), two glucan endo-1,3-beta-glucosidase (JK817631 and JK817632), a polygalacturonase (*PG*; JK817590), a expansion, (*EXP*; JK817639), a fasciclin-like arabinogalactan-protein (*FLA*; JK817668), a pectin methylesterase (*PME*; JK817660) and Xyloglucan galactosyltransferase (JK817677). Among them, *XTH9*, *EXP*, *PME* and *PRP2* genes were significantly down-regulated under B-deficiency stress at least in one time point of TO. While in CC, only *PME* and one of the *XTH9* (JK817615) genes were significantly reduced at 24 h and not changed at 6 and 12 h. *PG* gene was dramatically induced at 24 h in CC and 12 h in TO, respectively. In addition, the expression of *FLA* did not change significantly in TO, but it was significantly up-regulated by B-deficiency stress at 24 h in CC (Table 1).

**Table 1:** **List of boron-deficiency-responsive genes involved in cell wall metabolism under B-deficiency condition in citrus rootstock roots**.

**GeneBank no**.	**Putative function**	***e*-value**	**Carrizo citrange**			**Trifoliate orange**		
			**6h**	**12h**	**24h**	**6h**	**12h**	**24h**
JK817598	Xyloglucan endotransglucosylase/hydrolase protein 9, XTH9	2e-56	−1.34	−1.22	−1.75	−1.28	**−2.22**	**−2.08**
JK817599	Xyloglucan endotransglucosylase/hydrolase protein 9, XTH9	5e-53	−1.05	1.70	1.32	1.05	−1.27	**−2.13**
JK817606	Xyloglucan endotransglucosylase/hydrolase protein 9, XTH9	1e-129	1.01	1.05	1.00	−1.20	−1.09	**−2.22**
JK817615	Xyloglucan endotransglucosylase/hydrolase protein 9, XTH9	2e-50	−1.3	1.04	**−2.22**	1.07	**−2.38**	**−4.17**
JK817639	Expansion, EXP	6e-41	−1.25	−1.04	−1.37	1.03	−1.14	**−2.44**
JK817660	Pectin methylesterase, PME	1e-39	−1.42	−1.73	**−2.82**	−1.23	**−2.92**	−1.15
JK817590	Polygalacturonase, PG	2e-28	1.00	1.58	**2.60**	1.28	**2.02**	1.64
JK817668	Fasciclin-like arabinogalactan-protein, FLA	2e-19	1.49	1.17	**2.37**	1.27	−1.56	−1.69
JK817586	Proline-rich cell wall protein 2, PRP2	7e-19	1.17	1.47	1.29	−1.08	−1.43	−**2.04**
JK817604	Proline-rich cell wall protein 2, PRP2	2e-13	1.28	1.21	1.23	−**2.08**	−1.41	−**2.04**
JK817631	Glucan endo-1,3-beta-glucosidase	4e-80	0.76	**2.21**	**2.18**	**2.19**	**7.47**	1.43
JK817632	Glucan endo-1,3-beta-glucosidase	4e-64	1.15	**2.06**	**2.51**	**2.18**	**4.26**	1.38
JK817677	Xyloglucan galactosyltransferase	5e-70	1.05	**2.53**	**2.07**	1.30	1.34	1.39

Different expression levels based on fold change (FC, signal from B deficient roots/signal from B sufficient roots; “-” means the value of the signal from B sufficient roots/signal from B deficient roots) is indicated. Significant difference (FDR < 0.01 and fold change ≥ 2) in relative level are shown in bold.

### Transmembrane transporter related genes were modulated by B-deficiency

Plant cell membranes play critical roles in cell homeostasis, signal transduction, nutrition and stress responses. As expected, a large part of the up-regulated genes identified in this work is involved in transmembrane transport. Seventeen genes involving transmembrane transport were induced by B-deficiency treatment, including ten aquaporins (two NIP family aquaporins, six PIP family aquaporins, and two TIP family aquaporins), an ammonium transporter, three phosphate transporter, an ABC transporter C family, an annexin D1 and a voltage-dependent anion-selective channel (Table 2).

**Table 2:** **List of several boron-deficiency-responsive genes involved in transmembrane transport**.

**GeneBank no**.	**Putative function**	***e*-value**	**Carrizo citrange**			**Trifoliate orange**		
			**6h**	**12h**	**24h**	**6h**	**12h**	**24h**
JK817709	Aquaporin PIP1;1	1e-20	1.37	**2.32**	**2.34**	1.02	1.58	1.86
JK817714	Aquaporin PIP1;2	2e-50	1.68	**2.81**	**2.54**	0.75	1.46	1.55
JK817607	Aquaporin PIP1;3	1e-60	1.35	**2.03**	**3.01**	1.4	1.81	**2.25**
JK817645	Aquaporin PIP2;1	1e-80	1.55	1.52	**3.06**	1.09	1.58	1.54
JK817679	Aquaporin PIP2;2	3e-52	1.84	**2.48**	**2.01**	1.11	1.21	1.32
JK817635	Aquaporin PIP2;7	1e-54	1.4	1.05	**2.78**	1.09	1.94	1.54
JK817649	Aquaporin TIP2;2	1e-102	1.12	1.14	**3.10**	1.26	**2.56**	1.43
JK817676	Aquaporin TIP4;1	1e-39	1.62	1.86	**2.21**	1.05	1.31	1.16
JK817582	Aquaporin NIP5;1	3e-47	1.04	**5.20**	**4.66**	1.24	**3.81**	**2.35**
JK817718	Aquaporin NIP5;1	1e-45	1.32	**2.31**	**2.45**	1.05	**2.62**	1.33
JK817588	Phosphate transporter	5e-94	1.15	1.48	**2.12**	1.12	1.64	0.96
JK817627	Phosphate transporter	3e-84	1.06	1.94	**2.45**	1.59	**2.09**	1.22
JK817628	Phosphate transporter	2e-44	1.29	1.44	**2.27**	1.10	1.80	1.55
JK817610	Ammonium transporter	4e-53	1.07	1.10	**−2.70**	**−2.08**	1.22	−1.04
JK817658	ABC transporter C family	2e-21	−1.02	−1.20	**3.74**	−1.32	1.21	1.24
JK817688	Annexin D1	6e-19	1.41	**2.25**	1.38	1.81	1.57	**3.84**
JK817587	Voltage-dependent anion-selective channel	1e-45	1.26	1.94	**2.61**	**−2.7**	**2.04**	1.23

Different expression levels based on fold change (FC, signal from B deficient roots/signal from B sufficient roots; “-” means the value of the signal from B sufficient roots/signal from B deficient roots) is indicated. Significant difference (FDR < 0.01 and fold change ≥ 2) in relative level are shown in bold.

A very important gene belonging to the NIPs family, *NIP5;1* (JK81752 and JK817718), was identified to be differentially expressed under B-deficiency conditions. The expression of *NIP5;1* was up-regulated significantly by 12 h and 24 h B-deficiency stress and no change at 6 h in both CC and TO (Table 2). Six genes belonging to PIPs family were identified: *PIP1;1* (JK817709), *PIP1;2* (JK817714), *PIP1;3* (JK817607), *PIP2;1* (JK817645), *PIP2;2* (JK817679), and *PIP2;7* (JK817635). All the three genes (*PIP1;1, PIP1;2*, and *PIP1;3*) of PIP1 subfamily were dramatically induced in CC at both 12 h and 24 h under B-deficiency conditions, but only *PIP1;3* was induced significantly in TO at 24 h. As for the PIP2 subfamily genes (*PIP2;1, PIP2;2*, and *PIP2;7*), *PIP2;1*and *PIP2;7* were up-regulated after 24 h of B-deficiency stress in CC, while *PIP2;2* was induced after 12 h of B-deficiency stress remaining up-regulated at 24 h. By contrast, in TO, the expression of these three genes did not change significantly at any of the three time points. Two genes belonging to the TIPs family [*TIP2;2* (JK817649) and *TIP4;1* (JK817676)] were identified in this study. Both *TIP2;2* and *TIP4;1* were significantly up-regulated after 24 h under B-deficiency stress in CC, but in TO only *TIP2;2* was up-regulated at 12 h (Table 2).

In addition, several other types of transmembrane transporter were also identified in our array analysis, such as three phosphate transporters (JK817627, JK817628, and JK817610), and an ammonium transporter (JK817610) (Table 2).

### Genes involved in several metabolic pathways were changed in response to B-deficiency

In order to know which metabolic pathways were affected under B-deficiency, all the genes were analyzed according to the KEGG pathway database (KEGG=Kyoto Encyclopedia of Genes and Genomes; http://www.genome.jp/kegg/pathway.html). A total of 23 metabolic pathways were affected by B-deficiency, namely glycolysis/gluconeogenesis, phenylpropanoid biosynthesis, alanine, aspartate and glutamate metabolism, nitrogen metabolism, proteasome, peroxisome (Table 3 and Table S2).

**Table 3:** **List of boron-deficiency-responsive genes involved in three metabolic pathway according to the KEGG pathway database (KEGG: Kyoto Encyclopedia of Genes and Genomes; http://www.genome.jp/kegg/pathway.html)**.

**GeneBank no**.	**Putative function**	***e*-value**	**Carrizo citrange**			**Trifoliate orange**		
			**6h**	**12h**	**24h**	**6h**	**12h**	**24h**
**LIGNIN METABOLISM**								
JK817683	Phenylalanine ammonia-lyase, PAL	8e-57	1.13	1.61	1.33	**2.24**	**2.44**	**2.46**
JK817661	4-coumarate:CoA ligase, 4CL	1e-123	1.43	1.58	1.17	1.23	**2.09**	**2.69**
JK817644	Cinnamoyl-CoA reductase4, CCR4	1e-23	−1.49	−1.23	**−2.63**	1.28	**7.10**	**2.56**
JK817640	Peroxidase, POD	3e-34	1.24	1.20	**3.13**	−1.03	1.53	1.37
JK817712	Peroxidase, POD	6e-44	1.71	**2.27**	**3.11**	1.43	**3.15**	1.42
**NITROGEN METABOLISM**								
JK817620	Asparagine synthetase	2e-27	1.07	1.37	**2.83**	1.83	1.95	1.18
JK817610	Ammonium transporter	4e-53	1.07	1.10	**−2.70**	**−2.08**	1.22	−1.04
**GLYCOLYTIC PATHWAY**								
JK817680	2-phospho-D-glyceratehydrolase	6e-56	1.14	**2.09**	**2.29**	1.17	**2.36**	1.36
JK817717	Glyceraldehyde-3-phosphate dehydrogenase	3e-76	1.45	**2.28**	**4.23**	1.56	**2.86**	1.49

Different expression levels based on fold change (FC, signal from B deficient roots/signal from B sufficient roots; “-” means the value of the signal from B sufficient roots/signal from B deficient roots) is indicated. Significant difference (FDR < 0.01 and fold change ≥ 2) in relative level are shown in bold.

**Figure 6:**
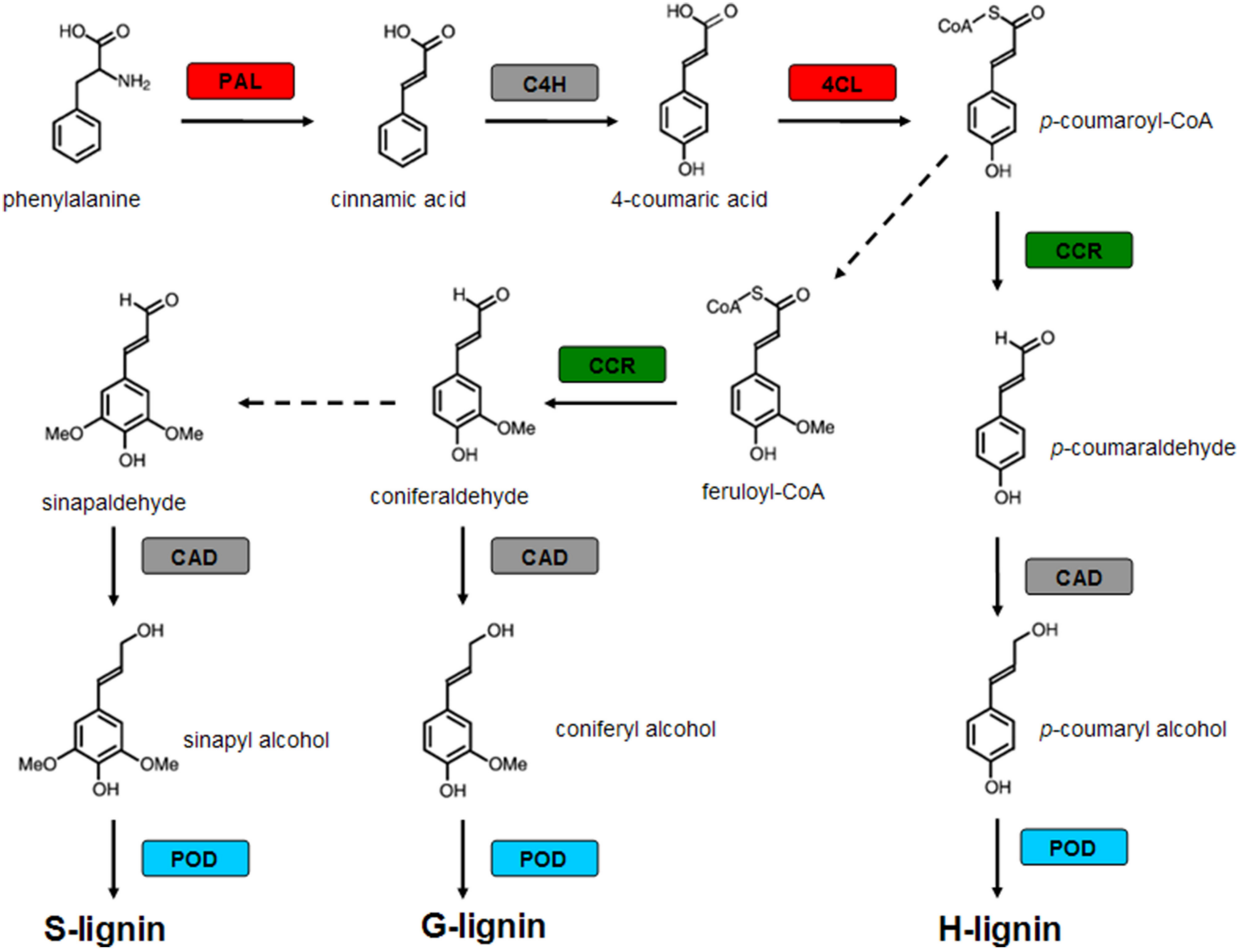
**Modulation of genes encoding enzymes involved in lignin biosynthetic pathway by boron (B)-deficiency stress**. Red boxes indicate genes up-regulated only in TO by B deficient stress, green boxes indicate genes down-regulated in CC and up-regulated in TO by B deficient stress, blue boxes indicate genes up-regulated both in CC and TO by B deficient stress, and gray boxes indicate genes not significantly modulated by B-deficiency stress. The dotted line means that some steps are not shown. PAL, Phenylalanine ammonia-lyase; C4H, cinnamate 4-hydroxylase; 4CL, 4-coumarate: CoA ligase; CCR, Cinnamoyl-CoA reductase; CAD, cinnamyl alcohol dehydrogenase; POD, peroxidase.

**Figure 7:**
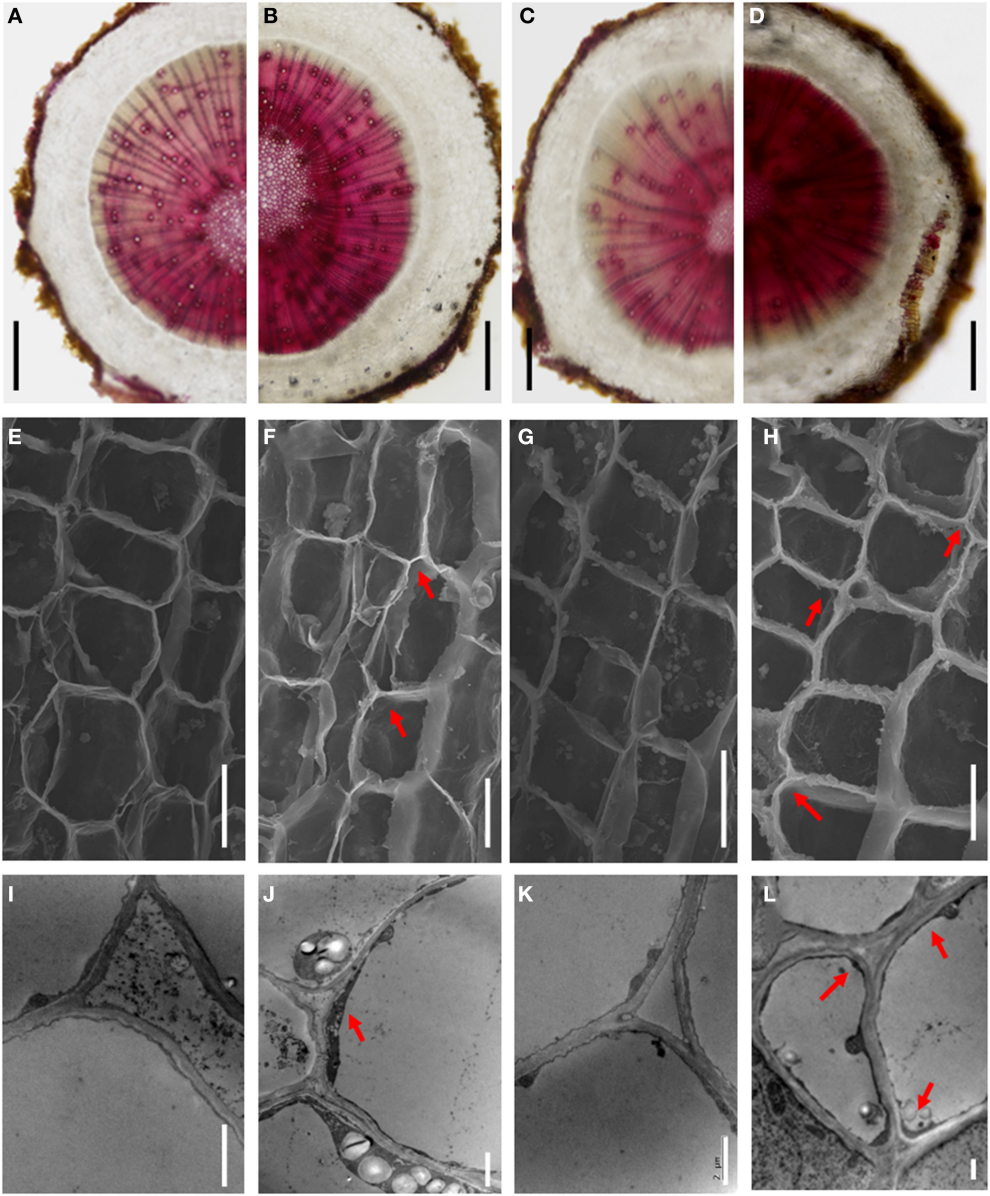
**The different morphology of the root cell walls between CC and TO under B-deficiency conditions. (A–D)** Phloroglucinol staining for lignin in root sections of CC and TO. **(E–H)** Scanning electron micrographs of transverse sections of CC and TO root. **(I–L)** Transmission electron micrographs of the root cell walls in CC and TO. Arrows show the thickened and folded walls. **(A,E,I)**, CC under normal conditions; **(B,F,J)**, CC under B-deficiency conditions; **(C,G,K)**, TO under normal conditions; **(D,H,L)**, TO under B-deficiency conditions. Bars = 2 mm in **(A–D)**, 10 μm in **(E–H)**, and 2 μm in **(I–L)**.

Four genes encoding key enzymes in the lignin biosynthesis pathway were significantly up-regulated under B-deficiency (Table 3 and Figure 6). These genes include phenylalanine ammonia-lyase (*PAL*; JK817683), 4-coumarate: CoA ligase (*4CL*; JK817661), cinnamoyl-CoA reductase4 (*CCR4*; KJ817664) and peroxidase (*POD*; JK817640 and JK817712). All these genes were up-regulated in TO plants root under B-deficiency at all time points, while only *POD* in CC plants root after 24 h B-deficiency treatment. In order to further investigate the different morphology of root cell wall induced by the change of lignin in the cell wall between CC and TO, histochemical staining and microscopy were performed. As shown in Figures 7A–D, the root sections were stained histochemically stained with phloroglucinol. According to the color intensity, which approximately reflects the total lignin content, the lignin quantity in the root cell walls of TO was much higher than CC under B-deficiency conditions (Figures 7B,D). Electron microscopy analysis demonstrated that the root of TO also showed heavily thickened cell walls (Figure 7H) and a thickened folded cell wall structure (Figure 7L) under B-deficiency conditions, compared with that of the control (Figures 7G,K). However, only a slight thickened cell walls were observed in CC (Figures 7E,F,I,J).

In addition, two genes, an asparagine synthetase gene (JK817620) and an ammonium transporter gene (JK817610), involved in the nitrogen metabolism were also identified in this work (Table 3). The expression of asparagine synthetase gene was up-regulated at 24 h under B-deficiency conditions, but distinct expression of ammonium transporter gene was found at that time point. Another two genes involved in glycolytic pathway were significantly affected by B-deficiency (Table 3), a glyceraldehyde-3-phosphatedehydrogenase (*GAPDH*; JK817717) and a 2-phospho-D-glyceratehydrolase (JK817680).

## Discussion

In this study, two citrus rootstocks, CC and TO, were used to further the identification genes responsive to B-deficiency using SSH and microarray analysis. Four SSH libraries were constructed with these two citrus rootstocks root tissue, then 7680 clones from the four SSH libraries were amplified and used for microarray analysis. A total of 139 unique genes that significantly changed (fold change ≥ 2 and FDR <0.01) upon 24 h B-deficiency stress, either in CC or TO, were identified. The microarray results of differential expression genes was further confirmed by quantitative real-time PCR.

### Phenotypic performance and citrus rootstocks B-deficiency tolerance

CC was reported as a B-deficiency tolerant rootstock (Sheng et al., 2009; Mei et al., 2011; Zhou et al., 2014). However, little is known about its genetic background. In the present work, its performance and response to B-deficiency were investigated, together with TO, a B-deficiency sensitive rootstock. In general, the symptoms of B-deficiency first appear in the growing regions of plants, and progress to the cessation of root elongation, reduced leaf expansion, and a loss of fertility (Marschner, 1995; Dell and Huang, 1997). In this work, the root, stem and leaf were affected under B-deficiency in both CC and TO, but the impact on TO was more serious than on CC in any part of the plants, especially in root (Figures 1A,B). In citrus, similar results have also been obtained in “Newhall” navel orange (*C. Sinensis* Osb. cv. Newhall) grafted on these two rootstocks (CC and TO) (Sheng et al., 2009).

Further study was performed on root physiology and morphology in both CC and TO. The results showed that B-deficiency caused a dramatic restriction on root growth, especially on root morphological traits of TO, whereas no effect was found on that of CC (Figures 2C,D,F) except for root volume (Figure 2E). This result suggested that the root of TO was more sensitive to B-deficiency than that of CC. Previous studies have shown that B-deficiency causes the cessation of root elongation in *A. thaliana* and other plants (Dell and Huang, 1997; Kato et al., 2009; Kocábek et al., 2009). This result was also similar to previous research on five citrus rootstocks by B-deficiency treatment (Mei et al., 2011). All these results supported that CC is more tolerant to B-deficiency than TO.

### Cell wall metabolism and citrus B-deficiency tolerance

It is known that plant cell wall is a complex and dynamic structure that is of fundamental importance in plant growth and development, and cell wall mediates the responses of plants to environmental and pathogen-induced stresses (Farrokhi et al., 2006). Since >90% of the B was found to be present in the water-insoluble fraction containing the cell walls in tobacco cells cultured under B-deficiency conditions (Matoh et al., 1992), B has been established as essential for cell wall structure and function (O'Neill et al., 2004). Beyond this, it has also been reported that the expression of several genes codifying for enzymes involved in cell wall metabolism were significantly changed in *Arabidopsis* roots under B-deficiency conditions (Camacho-Cristóbal et al., 2008), such as, xyloglucan endotransglycosylase/hydrolases (XTHs), expansins (EXP), pectin methylesterases (PME), and polygalacturonases (PGs). In this work, several genes encoding cell wall modifying enzymes were identified, and most of them were down-regulated by B-deficiency stress in both CC and TO (Table 1). The first group, JK817598, JK817599, JK817606, and JK817615 encodes XTH9, which catalyzes the transglycosylation of xyloglucan and has been proposed to be involved in the control of cell wall relaxation. Significant correlations between high levels of XTH activity and tissue elongation have been described in several cases (Schünmann et al., 1997; Burstin, 2000). Second, JK817639 encodes EXP, which is a plant cell wall proteins and participates in cell wall loosening (Cosgrove et al., 2002). Both genes responded to B-deficiency stress differently in TO than they did in the CC. The different responses to B-deficiency stress in CC and TO of these cell wall relative genes mentioned above indicated that they might contribute significantly to the B-deficiency tolerant nature of CC.

In addition, a dramatic morphological difference was found in root of TO between normal and B-deficiency treatment in this work (Figures 1B, 2) or in previous studies (Mei et al., 2011). It is known that root development and growth depend on cell division and expansion. Hence, this phenotypic difference can be explained by B-deficiency disrupting growing tissues through an effect on cell elongation (Brown et al., 2002), as B in cross-linking of cell wall RG-II and pectin assembly (Kobayashi et al., 1996; Matoh, 1997; Ishii and Matsunaga, 2001). In fact, RG II–borate complexes contribute significantly to the control of cell wall porosity (Fleischer et al., 1999) and tensile strength (Ryden et al., 2003). Considering the results here obtained, another possible explanation might be that the expression of several cell wall-modifying enzymes was decreased by B-deficiency stress (Table 1), which could inhibit the cell wall loosening (Cosgrove et al., 2002). Similar results were also reported by Camacho-Cristóbal et al. (2008) in Arabidopsis.

### Aquaporins and citrus B-deficiency tolerance

Aquaporins are water channel proteins of intercellular and plasma membranes which are involved in many functions of plants, such as nutrient acquisition, carbon fixation, cell signaling, and stress responses (Maurel, 2007). To date, two subgroups (NIPs and PIPs) of the MIP family have been reported to be involved in B transmembrane transport (Takano et al., 2006; Tanaka et al., 2008; Fitzpatrick and Reid, 2009). In our work, three different type aquaporins genes were regulated significantly under B-deficiency conditions: a NIPs, a TIPs, and a PIPs. This is the first report of a TIPs being up-regulated by B-deficiency stress (Table 2).

NIP5;1 is an efficient transmembrane transporter of B uptake and plant development under B limitation conditions have been reported in *Arabidopsis* (Takano et al., 2006). In this study, two *NIP5;1* genes were up-regulated in both CC and TO, especially in CC (Table 2). *NIP6;1* is another key gene of B transmembrane channel, which is a boric acid channel involved in preferential B transport to growing tissues of plants and showed the function of a boric acid channel in shoots in *Arabidopsis* (Tanaka et al., 2008). In this study, *NIP6;1* gene wasn't identified maybe because it is expressed in shoot not in root.

PIP and TIP aquaporins are similar to NIP in gene structure, so they may have similar function in plant's B transmembrane transport. Previous study has shown that a maize aquaporin, ZmPIP1, when expressed in *Xenopus* oocytes, could account for 25% of B uptake (Dordas et al., 2000). Recently, transport assays in yeast confirmed that two barley aquaporin, HvPIP1;3 and HvPIP1;4, are both capable of B transmembrane transport (Fitzpatrick and Reid, 2009). In this study, total six PIP and two TIP aquaporins were identified in both CC and TO, many of them were up-regulated under B-deficiency conditions (Table 2). In fact, PIPs aquaporin family can be further divided into two phylogenetic subgroups, PIP1 and PIP2. All the reported genes *ZmPIP1*, *HvPIP1;3* and *HvPIP1;4* belong to the PIP1 subgroup. In this study, two genes belong to PIP2 subgroup were also identified under B-deficiency condition, and up-regulated by B-deficiency stress. In the present work, two different type TIP aquaporins (TIP2;2 and TIP4;1) were also identified under B-deficiency, and their expression was up-regulated in both CC and TO. Recent studies have also reported that TIP5;1 is involved in B transport in *Arabidopsis* (Pang et al., 2010). These results showed that PIP and TIP aquaporins may have same function in plant B transmembrane transport. In this work, all the PIP and TIP aquaporins genes were up-regulated under B-deficiency conditions in CC, but only *PIP1;3* at 24 h and *TIP2;2* at 12 h were induced significantly by B-deficiency stress in TO. These results could explained why CC was more tolerance than TO to B-deficiency.

### Metabolic and citrus B-deficiency tolerance

To date, it has been reported that B involved in many functions including the formation of cell wall, sugar transport, cell wall synthesis and lignification, carbohydrate metabolism, RNA metabolism, respiration, indole acetic acid metabolism, phenol metabolism and membrane transport (Brown et al., 2002; Bolaños et al., 2004). Therefore, B has an influence on many metabolic pathways in plants. However, the mechanism of B involvement in many cases is not yet fully understood (Bolaños et al., 2004). In our work, a large number of differentially expressed genes identified in this study belong to the metabolism group. According to the KEGG pathway database (KEGG=Kyoto Encyclopedia of Genes and Genomes; http://www.genome.jp/kegg/pathway.html), a total of 23 metabolic pathways were affected by B-deficiency (Table 3 and Table S2), and some of these metabolic pathways have not previously been reported to be associated with B-deficiency stress.

In particular, the genes involved in the metabolic pathway of lignin biosynthesis were significantly affected by B-deficiency stress (Table 3 and Figure 6). It is known that lignin is a complex phenylpropanoid polymer mainly found in walls of xylem cells such as tracheary elements and xylary fibers (Boudet, 1998). Lignin is considered to be dehydrogenatively polymerized from the monolignols *p*-coumaryl alcohol, coniferyl alcohol, and sinapyl alcohol. These monolignols are synthesized through the general phenylpropanoid and monolignol-specific pathways (Figure 6), in these pathways phenylalanine ammonia-lyase (PAL), 4-coumarate:CoA ligase (4CL), cinnamoyl-CoA reductase (CCR) and peroxidase (POD) play a very important role. In our work, these 4 genes were significantly increased under B-deficiency conditions in the root of TO (Table 3 and Figure 6). These results indicated that B-deficiency has significant influence on lignin biosynthesis in plants, and it will cause lignification of root tip cells. In order to demonstrate this result, further investigation was carried on the root cell wall by histochemical staining and microscopy. As shown in Figure 7, the lignin quantity in the root cell walls of TO was significantly increased under B-deficiency conditions. On the other hand, the different morphology of root cell wall between CC and TO was also observed by Electron microscopy analysis. The root of TO showed heavily thickened cell walls (Figure 7H) and a thickened folded cell wall structure (Figure 7L) under B-deficiency conditions, compared with that of the control (Figures 7G,K). However, only a slight thickened cell walls were observed in CC (Figures 7E,F,I,J). These results not only indicated that B-deficiency significantly altered the lignin biosynthesis in citrus plants, but also indicated that lower lignification might contribute to CC being more tolerant than TO to B-deficiency stress. In addition, the ligneous cells of root tips will lose absorption and division functions. Thus, the root tips will stop development and form a swelling (Figure 1B). Previous work has proven that B involved in lignin metabolism (Ghanati et al., 2002; Bellaloui, 2012), and the lignin content increased in both B-deficiency and toxicity (Ruiz et al., 1998). It is known that lignin can lead to the loss of root B absorption capacity. Therefore, this seems to be a very important factor for the reason of CC is more tolerant to B-deficiency than TO.

Many research have reported that B is possible involve in nitrogen metabolism (Camacho-Cristóbal and González-Fontes, 2007; Matas et al., 2009; Beato et al., 2010). B-deficiency affects the nitrogen assimilation not only on the transcript level of related genes but also on the activity of related enzyme (Camacho-Cristóbal and González-Fontes, 1999, 2007). This decreased nitrate content was attributable to the lower net nitrate uptake rate found in B-deficient plants, probably as a consequence of the drop in the levels of root plasma membrane H+-ATPase (PMA2) transcript during the B-deficient treatment (Camacho-Cristóbal and González-Fontes, 2007). In this work we identified two genes involved in ammonium assimilation, One (JK817620) encodes asparagine synthetase gene, another one (JK817610) encodes ammonium transporter gene (Table 3). Asparagine is a primordial amino acid in the composition of xylem and phloem sap in vascular plants, and previous studies have reported that B-deficiency led to an increase in root asparagine content and a decline in glutamine synthetase activity, suggesting that B-deficiency may promote ammonium assimilation via asparagine synthetase in tobacco roots (Camacho-Cristóbal and González-Fontes, 2007). Further quantitative real-time PCR analyses showed that asparagine synthetase gene expression was increased under B-deficiency in tobacco roots (Camacho-Cristóbal and González-Fontes, 2007). In this study, the expression level of asparagine synthetase was significantly higher in CC at 24 h, but not in TO (Table 3). This result indicated that more nitrogen was absorbed via asparagine synthetase in CC root to maintain normal growth under B-deficiency conditions. In addition, in this work we also find that two key genes codifying enzymes involved in carbon metabolism were affected by B-deficiency (Table 3). It is known that nitrogen and carbon are two main structural matters in plant, so when their metabolism was affected by B-deficiency the biomass was also influenced. Thus, this result may explain the decrease of plant biomass in both CC and TO (Figures 1, 2A).

It is worthy to note that a large number of genes regulated by B-deficiency in this study encode unknown proteins; these genes may further influence the mechanism of the B-deficiency tolerance of citrus rootstock.

## Conclusion

In conclusion, B-deficiency treatment influenced significantly the growth and development, and B uptake in both CC and TO. However, CC as a B efficient rootstock, could grow better than TO under low B conditions. To understand the molecular basis of these different phenotypic performance to B-deficiency, SSH and microarray approaches were combined to identify the potential important or novel genes responsive to B-deficiency. A number of differentially expressed genes were identified in either CC or TO. Aquaporins family genes were up-regulated under B-deficiency conditions, especially in CC. Many genes involved in several metabolic pathways were differentially changed in CC, likely to adapt to B-deficiency stress. Cell wall-related genes were down regulated while lignin metabolism-related genes were up-regulated in TO under B-deficiency stress, possibly affecting the root elongation and B absorption. All these results indicated that CC was more tolerant than TO to B-deficiency stress. The B-deficiency responsive genes identified in this study could provide further information for understanding the mechanisms of B tolerance in citrus.

### Conflict of interest statement

The authors declare that the research was conducted in the absence of any commercial or financial relationships that could be construed as a potential conflict of interest.

## Abbreviations

B: Boron
SSH: Suppression subtractive hybridization
CC: Carrizo citrange
TO: Trifoliate orange
SIPs: Small basic intrinsic proteins
NIPs: Nodulin-26-like intrinsic protein
TIPs: Tonoplast intrinsic protein
PIPs: Plasma membrane intrinsic proteins
MIP: Major intrinsic protein
XTH: Xyloglucan endotransglycosylase/hydrolase
PRP: Proline-rich cell wall protein
PG: Polygalacturonases
EXP: Expansion
FLA: Fasciclin-like arabinogalactan-protein
PME: Pectin methylesterase
KEGG: Kyoto encyclopedia of genes and genomes
PAL: Phenylalanine ammonia-lyase
4CL: 4-Coumarate:CoA ligase
CCR: Cinnamoyl-CoA reductase
POD: Peroxidase.
