# Supplementary material for: Transcription profiles of boron-deficiency-responsive genes in citrus rootstock root by suppression subtractive hybridization and cDNA microarray
Source: Front Plant Sci. 2015 Jan 28;5:795. doi: 10.3389/fpls.2014.00795 (PMC4309116; doi:10.3389/fpls.2014.00795)
Supplement: Supplementary file 1 [file Table1.DOC]

**SUPPLEMENTARY MATERIAL FILE 1**

**APPENDIX S1 ∣ Primers used for amplifying selected and control genes for real-time PCR.**

| **GeneBank no.** | **Putative function** | **Primer sequences (5' to 3')** |
| --- | --- | --- |
| JK817631 | Beta-1,3-glucanase | F-GAAGTCTCTTTGACGCCATCT/R-ACCCCCGCCGGTTTTCTG |
| JK817608 | Elongation factor 1-alpha | F-TCTCGAACTTCCACAAGGCAAT/R-GGGTGCTCGACAAGCTCAA |
| JK817604 | Proline-rich cell wall protein | F-CCGCCTGGATTCCAGTGT/R-ACAAGTCGCATTCATCAATTGC |
| JK817615 | Xyloglucan endotransglucosylase/hydrolase | F-TGCTGCATTTTTTGGCATTATC/R-GGCTTTGAAATTGATGCATGTG |
| JK817695 | Vinorine synthase | F-TCTACGGCAGAGAATGAATCCA/R-CACCAATCCCCCGGATATTA |
| JK817609 | Hypothetical protein | F-GGGAGGCAGCAGTGAGGAA/R-CACGCGACTTGGCTGGTT |
| JK817678 | Isoflavone 2'-hydroxylase | F-GGAATGCCTGAAAGCTCAAATT/R-CGTCCTGATCCAGGTGTACGA |
| JK817629 | Isoflavone reductase | F-GCCAGCCTGGAGTGAGTGTT/R-AAACGGCCTTAGCATTTCCAT |
| JK817640 | Peroxidase | F-GGATGGTCCAGGGATTTGG/R-GGCCGGCGAGATGCA |
| JK817594 | Elongation factor EF-1 | F-CCTGGGTGCTCGACAAACTC/R-CGAACTTCCACAAGGCAATATC |
| Control gene | β-Actin | F-CCGACCGTATGAGCAAGGAAA/R-TTCCTGTGGACAATGGATGGA |
