# Supplementary material for: Transcription profiles of boron-deficiency-responsive genes in citrus rootstock root by suppression subtractive hybridization and cDNA microarray
Source: Front Plant Sci. 2015 Jan 28;5:795. doi: 10.3389/fpls.2014.00795 (PMC4309116; doi:10.3389/fpls.2014.00795)
Supplement: Supplementary file 2 [file Table2.DOC]

**SUPPLEMENTARY MATERIAL FILE 2**

**APPENDIX S2 ∣** **A complete list of differentially expressed genes respond to boron deficiency stress in Carrizo citrange and Trifoliate orange. Different expression levels based on fold change (FC, signal from B deficient roots/signal from B sufficient roots; ‘-’ means the value of the signal from B sufficient roots/signal from B deficient roots) is indicated. Significant differnce (FDR test, p<0.01) in relative level is shown in bold.**

| **GeneBank**  **no.** | **Putative function** | **e-**  **value** | **Carrizo citrange** | | | **Trifoliate orange** | | |
| --- | --- | --- | --- | --- | --- | --- | --- | --- |
|  |  |  | **6h** | **12h** | **24h** | **6h** | **12h** | **24h** |
| **1 Metabolism** | | | | | | | | |
| JK817683 | Phenylalanine ammonia-lyase, PAL | 8e-57 | 1.13 | 1.61 | 1.33 | **2.24** | **2.44** | **2.46** |
| JK817661 | 4-coumarate:CoA ligase, 4CL | 1e-123 | 1.43 | 1.58 | 1.17 | 1.23 | **2.09** | **2.69** |
| JK817644 | Cinnamoyl-CoA reductase4, CCR4 | 1e-23 | -1.49 | -1.23 | **-2.63** | 1.28 | **7.10** | **2.56** |
| JK817640 | Peroxidase, POD | 3e-34 | 1.24 | 1.20 | **3.13** | -1.03 | 1.53 | 1.37 |
| JK817712 | Peroxidase, POD | 6e-44 | 1.71 | **2.27** | **3.11** | 1.43 | **3.15** | 1.42 |
| JK817601 | Isoflavone reductase, IRL | 1e-124 | 1.51 | 1.56 | **2.01** | -1.39 | 1.84 | 1.06 |
| JK817629 | Isoflavone reductase, IRL | 2e-74 | **3.03** | **2.76** | **2.20** | 1.33 | 1.47 | 1.61 |
| JK817678 | Isoflavone 2'-hydroxylase | 5e-33 | 1.09 | **2.15** | **2.96** | 1.38 | 1.11 | 1.34 |
| JK817680 | 2-phospho-D-glyceratehydrolase | 6e-56 | 1.14 | **2.09** | **2.29** | 1.17 | **2.36** | 1.36 |
| JK817717 | Glyceraldehyde-3-phosphate dehydrogenase | 3e-76 | 1.45 | **2.28** | **4.23** | 1.56 | **2.86** | 1.49 |
| JK817664 | Lysophosphatidic acid acyltransferase | 7e-16 | 1.09 | 1.43 | **2.04** | 1.09 | 1.29 | 1.39 |
| JK817667 | Lipase | 9e-41 | 1.01 | -1.26 | **-2.13** | -1.33 | 1.37 | 1.24 |
| JK817620 | Asparagine synthetase | 2e-27 | 1.07 | 1.37 | **2.83** | 1.83 | 1.95 | 1.18 |
| JK817711 | 5-methyltetrahydropteroyltriglutamate-homocysteine methyltransferase | 1e-107 | **2.02** | **2.48** | **2.09** | 1.18 | **2.24** | **2.41** |
| JK817699 | S-adenosyl-L-homocysteine hydrolase | 6e-14 | 1.52 | 1.95 | **2.36** | 1.08 | 1.69 | 1.35 |
| **2 Energy** | | | | | | | | |
| JK817666 | Adenosine diphosphatase | 1e-18 | 1.03 | 1.36 | **2.33** | 1.14 | 1.42 | 1.13 |
| JK817669 | Purine permease | 2e-15 | 1.05 | 1.38 | **2.48** | 1.28 | 1.50 | 1.31 |
| JK817690 | ADP,ATP carrier protein | 6e-83 | 1.03 | **2.79** | **2.06** | 1.59 | **4.12** | 1.49 |
| **3 Transcription and signal transduction** | | | | | | | | |
| JK817701 | Homeobox-leucine zipper protein | 1e-128 | 1.53 | 1.40 | **2.99** | 1.24 | **2.51** | -1.08 |
| JK817702 | Homeobox-leucine zipper protein | 2e-86 | 1.06 | 1.44 | **2.10** | 1.06 | 1.27 | 1.21 |
| JK817705 | Receptor protein kinase | 2e-70 | 1.4 | 1.56 | **2.35** | 1.33 | 1.82 | 1.05 |
| JK817585 | DELLA domail GRAS family transcription factor | 2e-24 | 1.03 | 1.84 | **2.18** | 1.21 | 1.43 | 1.35 |
| JK817624 | DELLA domail GRAS family transcription factor | 5e-25 | 1.07 | -1.10 | **2.16** | 1.24 | 1.51 | 1.33 |
| JK817637 | Transcription factor | 3e-49 | 1.12 | **2.23** | **2.36** | 1.31 | 1.80 | 1.31 |
| JK817656 | Cleavage and polyadenylation specificity factor | 1e-118 | -1.79 | -1.56 | **-4.55** | 1.25 | **4.55** | 1.80 |
| JK817653 | Signal peptide peptidase-like 2C | 1e-42 | 1.17 | **2.01** | **2.31** | 1.24 | 1.63 | 1.21 |
| JK817687 | Prenylated Rab acceptor protein | 1e-32 | 1.06 | **2.44** | 1.76 | -1.15 | 1.35 | **-2.33** |
| JK817682 | Rhicadhesin receptor protein | 1e-42 | 1.17 | **2.68** | **3.19** | **-3.13** | **2.39** | **2.23** |
| JK817634 | Phosphatase 2C | 1e-60 | 1.03 | **-2.08** | **-2.32** | 1.32 | 1.06 | 1.41 |
| JK817648 | Mitogen-activated protein kinase, (MAPK) | 0 | 1.5 | **2.86** | **3.28** | 1.78 | **4.98** | 1.63 |
| **4 Subcellular localization** | | | | | | | | |
| JK817598 | Xyloglucan endotransglucosylase/  hydrolase protein 9, XTH9 | 2e-56 | -1.34 | -1.22 | -1.75 | -1.28 | **-2.22** | **-2.08** |
| JK817599 | Xyloglucan endotransglucosylase/  hydrolase protein 9, XTH9 | 5e-53 | -1.05 | 1.70 | 1.32 | 1.05 | -1.27 | **-2.13** |
| JK817606 | Xyloglucan endotransglucosylase/  hydrolase protein 9, XTH9 | 1e-129 | 1.01 | 1.05 | 1.00 | -1.2 | -1.09 | **-2.22** |
| JK817615 | Xyloglucan endotransglucosylase/  hydrolase protein 9, XTH9 | 2e-50 | -1.3 | 1.04 | **-2.22** | 1.07 | **-2.38** | **-4.17** |
| JK817586 | Proline-rich cell wall protein 2, PRP2 | 7e-19 | 1.17 | 1.47 | 1.29 | -1.08 | -1.43 | **-2.04** |
| JK817604 | Proline-rich cell wall protein 2, PRP2 | 2e-13 | 1.28 | 1.21 | 1.23 | **-2.08** | -1.41 | **-2.04** |
| JK817639 | Expansin | 6e-41 | -1.25 | -1.04 | -1.37 | 1.03 | -1.14 | **-2.44** |
| JK817590 | polygalacturonase | 2e-28 | 1 | 1.58 | **2.60** | 1.28 | **2.02** | 1.64 |
| JK817660 | Pectin methylesterase, PME | 1e-39 | -1.42 | -1.73 | **-2.82** | -1.73 | **-2.92** | -1.15 |
| JK817631 | Glucan endo-1,3-beta-glucosidase | 4e-80 | 0.76 | **2.21** | **2.18** | **2.19** | **7.47** | 1.43 |
| JK817632 | Glucan endo-1,3-beta-glucosidase | 4e-64 | 1.15 | **2.06** | **2.51** | **2.18** | **4.26** | 1.38 |
| JK817677 | Xyloglucan galactosyltransferase | 5e-70 | 1.05 | **2.53** | **2.07** | 1.3 | 1.34 | 1.39 |
| JK817693 | Tubulin alpha chain | 2e-82 | 1.38 | 1.81 | **2.81** | 1.21 | 1.94 | 1.69 |
| JK817697 | Tbulin beta chain | 4e-59 | 1.08 | -1.05 | **-2.13** | -1.69 | 1.15 | -1.20 |
| **5 Protein with binding function or cofactor requirement** | | | | | | | | |
| JK817654 | Lipid binding protein | 1e-39 | 1.02 | 1.67 | **2.05** | 1.15 | **2.04** | 1.25 |
| JK817665 | DNA binding protein | 5e-42 | 1.26 | 1.45 | **2.25** | 1.23 | 1.23 | 1.11 |
| JK817691 | RNA-binding protein | 4e-57 | -1.28 | -1.39 | **-2.44** | -1.18 | -1.01 | -1.33 |
| **6 Cellular transport, transport facilitation and transport routes** | | | | | | | | |
| JK817709 | Aquaporin PIP1;1 | 1e-20 | 1.37 | **2.32** | **2.34** | 1.02 | 1.58 | 1.86 |
| JK817714 | Aquaporin PIP1;2 | 2e-50 | 1.68 | **2.81** | **2.54** | 0.75 | 1.46 | 1.55 |
| JK817607 | Aquaporin PIP1;3 | 1e-60 | 1.35 | **2.03** | **3.01** | 1.4 | 1.81 | **2.25** |
| JK817645 | Aquaporin PIP2;1 | 1e-80 | 1.55 | 1.52 | **3.06** | 1.09 | 1.58 | 1.54 |
| JK817679 | Aquaporin PIP2;2 | 3e-52 | 1.84 | **2.48** | **2.01** | 1.11 | 1.21 | 1.32 |
| JK817635 | Aquaporin PIP2;7 | 1e-54 | 1.4 | 1.05 | **2.78** | 1.09 | 1.94 | 1.54 |
| JK817649 | Aquaporin TIP2;2 | 1e-102 | 1.12 | 1.14 | **3.10** | 1.26 | **2.56** | 1.43 |
| JK817676 | Aquaporin TIP4;1 | 1e-39 | 1.62 | 1.86 | **2.21** | 1.05 | 1.31 | 1.16 |
| JK817582 | Aquaporin NIP5;1 | 3e-47 | 1.04 | **5.20** | **4.66** | 1.24 | **3.81** | **2.35** |
| JK817718 | Aquaporin NIP5;1 | 1e-45 | 1.32 | **2.31** | **2.45** | 1.05 | **2.62** | 1.33 |
| JK817588 | Phosphate transporter | 5e-94 | 1.15 | 1.48 | **2.12** | 1.12 | 1.64 | 0.96 |
| JK817627 | Phosphate transporter | 3e-84 | 1.06 | 1.94 | **2.45** | 1.59 | **2.09** | 1.22 |
| JK817628 | Phosphate transporter | 2e-44 | 1.29 | 1.44 | **2.27** | 1.1 | 1.80 | 1.55 |
| JK817610 | Ammonium transporter | 4e-53 | 1.07 | 1.10 | **-2.70** | **-2.08** | 1.22 | -1.04 |
| JK817658 | ABC transporter C family | 2e-21 | -1.02 | -1.20 | **3.74** | -1.32 | 1.21 | 1.24 |
| JK817688 | Annexin D1 | 6e-19 | 1.41 | **2.25** | 1.38 | 1.81 | 1.57 | **3.84** |
| JK817587 | Voltage-dependent anion-selective channel | 1e-45 | 1.26 | 1.94 | **2.61** | **-2.7** | **2.04** | 1.23 |
| **7 Cell rescue, defense and virulence** | | | | | | | | |
| JK817584 | Metallothionein-like protein | 2e-13 | 0.98 | 1.96 | **2.07** | 0.95 | **2.03** | 1.09 |
| JK817603 | Metallothionein-like protein | 1e-13 | 1.03 | **2.14** | 1.69 | 1.05 | **2.21** | 1.26 |
| JK817597 | Metallothionein-like protein | 1e-12 | 1.11 | 1.42 | 1.58 | 1.05 | **2.21** | 1.26 |
| JK817641 | Metallothionein-like protein | 1e-13 | 1.01 | **2.05** | **2.31** | 1.06 | **2.56** | 1.18 |
| JK817643 | Chitinase | 3e-39 | 1.22 | **2.38** | **2.34** | 1.54 | 1.69 | 1.32 |
| JK817647 | Chitinase | 3e-73 | 1.22 | **2.67** | **2.21** | 1.15 | **2.24** | 1.39 |
| JK817630 | Heat shock-related protein | 4e-33 | 1.76 | **-2.04** | 1.98 | -1.15 | **-2.86** | **-2.17** |
| JK817618 | DnaJ protein | 5e-29 | **-2.08** | -1.15 | -1.89 | **-2** | -1.16 | **-2.04** |
| JK817713 | Proteasome subunit alpha type | 1e-42 | 1.5 | 1.65 | **3.60** | **-2.78** | **2.15** | **2.64** |
| JK817668 | Fasciclin-like arabinogalactan-protein, FLA | 2e-19 | 1.49 | 1.17 | **2.37** | 1.27 | -1.56 | -1.69 |
| JK817617 | Peroxisomal(S)-2-hydroxy-acid oxidase | 2e-90 | 1.33 | **2.39** | **4.25** | 1.55 | 1.57 | 1.52 |
| JK817621 | BAHD acyltransferase | 1e-89 | 1.16 | **2.03** | **2.61** | 1.18 | **3.06** | 1.58 |
| JK817652 | Auxin-induced protein | 2e-43 | 1.15 | **2.13** | **2.20** | **2.45** | **2.10** | 1.62 |
| JK817673 | Senescence-associated protein | 9e-45 | -1.16 | **-1.59** | **-2.04** | -1.06 | -1.30 | -1.25 |
| JK817674 | SUMO-conjugating enzyme | 3e-59 | -1.69 | **1.24** | **2.99** | -1.47 | 1.21 | -1.14 |
| JK817675 | Defensin-like protein | 4e-12 | -1.3 | **3.12** | **2.12** | 1.26 | 1.34 | 1.13 |
| JK817651 | Glutathione-s-transferase | 3e-73 | -1.34 | **2.17** | **3.41** | **-2.38** | **2.29** | -1.12 |
| **8 Protein fate** | | | | | | | | |
| JK817681 | F-box family protein | 7e-26 | 1.11 | 1.45 | **2.36** | 1.38 | 1.11 | 1.07 |
| JK817670 | Ein3-binding F-box protein | 5e-29 | 1.01 | -1.28 | **-2.44** | 1.50 | 1.20 | 1.09 |
| JK817613 | Ubiquitin 10.2 | 1e-180 | -1.35 | 1.52 | **-2.38** | 1.33 | -1.18 | -1.56 |
| JK817703 | Ubiquitin-protein ligase | 4e-15 | 1.19 | 1.45 | **-2.22** | 1.52 | 1.13 | 1.26 |
| JK817700 | E3 ubiquitin-protein ligase | 9e-45 | 1.1 | 1.24 | **2.24** | 1.50 | 1.09 | 1.05 |
| **9 Protein synthesis** | | | | | | | | |
| JK817608 | Elongation factor 1-alpha | 7e-90 | 1.19 | **4.69** | **4.97** | **2.46** | **3.33** | 1.50 |
| JK817594 | Elongation factor 1-alpha | 3e-42 | 1.15 | **4.37** | **3.62** | **2.65** | **4.54** | 1.57 |
| JK817707 | 60S ribosomal protein L4 | 6e-53 | 1.26 | 1.73 | **2.94** | **-2.7** | **2.69** | 1.21 |
| JK817696 | 60S ribosomal protein L9 | 4e-95 | 1.2 | 1.51 | **3.22** | 1.11 | **2.23** | 1.32 |
| JK817708 | 60S ribosomal protein L12 | 1e-56 | 1.18 | **2.21** | 1.72 | **1.14** | 1.80 | **-2.13** |
| JK817715 | 60S ribosomal protein L13 | 1e-40 | 1.25 | **2.52** | **3.08** | 1.45 | **2.03** | 1.14 |
| JK817623 | 60S ribosomal protein L23 | 2e-49 | 1.18 | 1.69 | **2.10** | 1.69 | 1.68 | 1.06 |
| JK817650 | 60S ribosomal protein L26 | 2e-30 | 1.04 | 1.50 | **2.02** | 1.16 | **2.13** | 1.24 |
| JK817612 | 60S ribosomal protein L27 | 5e-32 | 1.16 | 1.87 | **2.07** | 1.38 | **2.68** | 1.15 |
| JK817642 | ribonuclease H protein | 2e-71 | -1.23 | -1.47 | **-2.33** | -1.12 | -1.05 | -1.14 |
| **10 Unclassified** | | | | | | | | |
| JK817581 | 3'-N-debenzoyl-2'-deoxytaxol N-benzoyl transferase | 1e-27 | 1.53 | 1.54 | **2.80** | 1.34 | **3.10** | 1.74 |
| JK817596 | 3'-N-debenzoyl-2'-deoxytaxol N-benzoyl transferase | 8e-52 | -1.12 | 1.07 | **-2.13** | **-2.08** | 1.43 | -1.02 |
| JK817605 | Bifunctional 3'-phosphoadenosine 5'-phosphosulfate synthase | 3e-36 | -1.04 | **2.20** | **2.37** | **-2.13** | **2.16** | 1.44 |
| JK817611 | 4,5-DOPA dioxygenase extradiol-like protein | 6e-20 | 1.19 | 1.67 | 1.29 | 1.21 | 1.02 | **2.65** |
| JK817622 | Pollen-specific protein | 3e-09 | 1.41 | **2.12** | **2.34** | **-3.85** | 1.98 | **2.52** |
| JK817626 | S locus protein 11 | 1e-41 | 1.19 | **2.06** | **2.92** | -1.59 | 1.58 | 1.35 |
| JK817685 | Mitochondrial processing peptidase | 3e-25 | 1.14 | 1.60 | **2.32** | 1.22 | 1.65 | 1.40 |
| JK817646 | Adenosylhomocysteinase | 1e-120 | 1.6 | **2.58** | **3.16** | **-2.33** | 1.87 | 1.92 |
| JK817692 | coated vesicle membrane protein, | 4e-41 | 1.02 | **1.57** | **2.05** | 1.35 | 1.29 | 1.15 |
| JK817694 | Germin-like protein subfamily 2 member 4 | 9e-50 | 1.32 | **2.41** | 1.34 | 1.13 | **1.21** | **2.94** |
| JK817695 | Vinorine synthase | 4e-20 | **2.53** | **2.34** | **2.55** | 1.11 | 1.39 | 1.35 |
| JK817706 | serine-rich protein-related | 5e-22 | -1.39 | -1.49 | **-2.27** | -1.16 | -1.27 | -1.23 |
| **11 Unknown** | | | | | | | | |
| JK817589 | Hypothetical protein | 9e-11 | -1.08 | -1.22 | **2.68** | -1.16 | -1.02 | 1.17 |
| JK817591 | Hypothetical protein | 2e-10 | 1.17 | 1.32 | **2.12** | -1.32 | -1.32 | 1.00 |
| JK817592 | Hypothetical protein | 2e-09 | 1.09 | 1.62 | **2.20** | -1.72 | 1.38 | 1.18 |
| JK817593 | Hypothetical protein | 5e-24 | -1.15 | 1.23 | **-2.13** | -1.69 | 1.52 | -1.23 |
| JK817609 | Hypothetical protein | 6e-30 | 1.12 | 1.31 | **2.86** | -1.39 | 1.66 | 1.46 |
| JK817614 | Hypothetical protein | 7e-42 | 1 | **2.22** | **3.04** | **-2.13** | **2.56** | 1.34 |
| JK817619 | Hypothetical protein | 1e-17 | 1.65 | **3.48** | **2.18** | **18.6** | 1.32 | 1.62 |
| JK817633 | Hypothetical protein | 3e-28 | -1.04 | **5.02** | **4.66** | 1.24 | **3.18** | **2.35** |
| JK817655 | Hypothetical protein | 2e-25 | 1.1 | 1.37 | **2.18** | -1.22 | 1.15 | 1.34 |
| JK817657 | Hypothetical protein | 1e-59 | 1.38 | **2.49** | **2.06** | 1.3 | **2.41** | 1.41 |
| JK817659 | Hypothetical protein | 7e-43 | 1.02 | **2.10** | 1.74 | 1.47 | 1.25 | **2.79** |
| JK817662 | Hypothetical protein | 9e-32 | **2.4** | **3.36** | 1.74 | -1.92 | -1.11 | **2.40** |
| JK817698 | Hypothetical protein | 5e-42 | 1.12 | 1.37 | **2.46** | 1.1 | 1.36 | 1.14 |
| JK817704 | Hypothetical protein | 1e-22 | 1.17 | 1.47 | **2.20** | 1.19 | 1.69 | 1.37 |
| JK817580 | Predicted protein | 5e-17 | 1 | 1.18 | **2.09** | 1.07 | 1.83 | 1.22 |
| JK817583 | predicted protein | 2e-41 | **-2.78** | 1.65 | 1.36 | 1.61 | **2.61** | **4.40** |
| JK817600 | Predicted protein | 7e-27 | 1.68 | **2.81** | 1.60 | -2.38 | **1.54** | **2.05** |
| JK817616 | Predicted protein | 3e-50 | -1.15 | -1.39 | **-2.33** | -1.64 | 1.52 | -1.39 |
| JK817625 | Predicted protein | 4e-09 | 1.04 | 1.39 | **2.47** | 1.09 | 1.69 | 1.15 |
| JK817638 | Predicted protein | 2e-44 | **2.59** | **-3.70** | **-4.76** | 1.91 | **2.48** | **3.22** |
| JK817636 | Predicted protein | 3e-35 | 1.32 | **2.13** | **2.54** | 1.05 | **2.26** | 1.33 |
| JK817663 | Predicted protein | 6e-06 | -1.15 | -1.53 | **-2.04** | 1.19 | 1.04 | -1.30 |
| JK817689 | Predicted protein | 1e-10 | 1.08 | **1.42** | **2.05** | -1.61 | 1.08 | 1.26 |
| JK817710 | Predicted protein | 6e-52 | 1.61 | **2.24** | 1.70 | -1.18 | **1.19** | **2.19** |
| JK817684 | Predicted protein | 7e-73 | 1.11 | **2.50** | 1.60 | 1.96 | **1.28** | **2.02** |
| JK817716 | Predicted protein | 9e-80 | 1.04 | 1.60 | **3.55** | 1.21 | **2.28** | 1.05 |
| JK817595 | Unnamed protein product | 2e-20 | 1.11 | 1.38 | **2.17** | 1.15 | **2.03** | 1.59 |
| JK817602 | Unnamed protein product | 6e-19 | 1.03 | 1.63 | **2.47** | 1.52 | **2.22** | 1.90 |
| JK817671 | Unnamed protein product | 3e-21 | 1.14 | 1.34 | **4.90** | 1.08 | 1.71 | 1.31 |
